# Combined Effects of Physical Activity and Diet on Cancer Patients: A Systematic Review and Meta-Analysis

**DOI:** 10.3390/nu16111749

**Published:** 2024-06-02

**Authors:** Petros C. Dinas, on behalf of the Students of Module 5104 (Introduction to Systematic Reviews), Marianthi Karaventza, Christina Liakou, Kalliopi Georgakouli, Dimitrios Bogdanos, George S. Metsios

**Affiliations:** 1 Department of Nutrition and Dietetics, School of Physical Education, Sport Science and Dietetics, University of Thessaly, 42130 Trikala, Greece; aikmakri@uth.gr (on behalf of the Students of Module 5104 (Introduction to Systematic Reviews)); markaraventza@gmail.com (M.K.); kgeorgakouli@uth.gr (K.G.); g.metsios@uth.gr (G.S.M.); 2 FAME Laboratory, School of Physical Education, Sport Science and Dietetics, University of Thessaly, 42131 Trikala, Greece; 3 School of Physical Education, Sport Science and Dietetics, University of Thessaly, 42131 Trikala, Greece; cliakou@uth.gr; 4 Department of Internal Medicine, University Hospital of Larissa, Faculty of Medicine, School of Health Sciences, University of Thessaly, 41110 Larissa, Greece; bogdanos@uth.gr

**Keywords:** nutrition, exercise, cancer indices

## Abstract

Background: The purpose of our systematic review was to examine the effects of any physical activity/exercise intervention combined with any diet/nutrition intervention on any biological/biochemical index, quality of life (QoL), and depression in breast, lung, colon and rectum, prostate, stomach, and liver cancer patients and/or cancer survivors. Methods: A systematic review and meta-analysis were undertaken, using PRISMA guidelines and the Cochrane Handbook. The systematic review protocol can be found in the PROSPERO database; registration number: CRD42023481429. Results: We found moderate-quality evidence that a combined intervention of physical activity/exercise and nutrition/diet reduced body mass index, body weight, fat mass, insulin, homeostatic model assessment for insulin resistance, C-reactive protein, triglycerides, and depression, while it increased high-density lipoprotein, the physical component of QoL, and general functional assessment of cancer therapy. Conclusions: We conclude that a combined intervention of physical activity/exercise and diet/nutrition may decrease body weight, fat mass, insulin levels, and inflammation, and improve lipidemic profile, the physical component of QoL, and depression in cancer patients and survivors. These outcomes indicate a lower risk for carcinogenesis; however, their applicability depends on the heterogeneity of the population and interventions, as well as the potential medical treatment of cancer patients and survivors.

## 1. Introduction

Cancer is a group of diseases that can affect almost all organs and tissues in the human body. It is characterized by the rapid creation of abnormal cells that grow unusually and spread around the body, which causes a cascade of unfavorable health effects [1]. Potential reasons for the development of cancer cells include exposure to ultraviolet and ionizing radiation, certain chemicals (e.g., asbestos, tobacco smoke, and alcohol), nutritional contaminants, and certain infections [1]. The most common types of cancers that appear in humans are breast, lung, colorectal, prostate, skin, and stomach cancers [1]. Cancer is a leading cause of human deaths worldwide, with approximately 10 million deaths in 2020; the most lethal cancers are lung, colon and rectum, liver, stomach, and breast cancer [1].

Cancer is also a major health burden, given its remarkable physical, emotional, and financial stress on patients, communities, and national health systems, especially those in low-income countries [1]. Recent evidence from 29 types of cancers and 204 countries and territories shows that the economic cost of cancers between 2020 and 2050 will be USD 25.2 trillion [2]. This is a substantial burden for national health systems, indicating the need for countermeasures to prevent and treat cancer. To date, several initiatives have been undertaken to reduce the cancer health burden and subsequently economic cost, such as the United Nations Sustainable Development Goal target 3.4 [3], the European Code Against Cancer [4], and the World Health Organization (WHO) Global Action Plan for the Prevention and Control of Noncommunicable Diseases [5].

The main pillars to consider for cancer prevention are as follows: (i) factors of cancer risk, namely smoking and tobacco use, infections, radiation, immunosuppressive medicines, and organ transplant; and (ii) factors that may pose a risk of cancer, namely nutrition, alcohol, physical inactivity, obesity, diabetes, and environmental factors [6]. Evidence on the contribution of several diets to cancer development is controversial, given that there are foods that reduce the risk of cancer and foods that increase the risk of cancer [7]. For instance, consumption of >1 sugar-sweetened beverage per day increases the incidence of liver cancer [8], while consumption of green-yellow and cruciferous vegetables reduces cancer risk [9]. Furthermore, evidence from systematic reviews [10,11,12] showed small or uncertain effects of red meat consumption, or a reduction in its consumption, on cancer prevalence and mortality. Also, there is no association between animal protein or plant protein consumption and cancer mortality [13]. Alcohol consumption is associated with an increased risk of breast cancer, whereas consumption of fruits and vegetables is inversely associated with lung cancer [7]. Regarding physical activity, there is solid evidence that increased levels are associated with a 20% reduced risk of breast cancer and a 40–50% reduced risk of colorectal cancer [7]. This evidence suggests that physical activity is a very promising intervention to reduce cancer incidence. Cancer patients also have a poor quality of life (QoL) [14] and depression [15]. It was found that only 5.02% of cancer patients displayed high QoL and 12.54% displayed average QoL [14]. Evidence has shown that increased physical activity reduces depression levels, whereas high intensities of physical activity cause euphoria due to increased production of β-endorphin in the brain, indicating decreased levels of anxiety and depression [16].

There is a growing interest in exploring the effects of combined dietary and physical activity interventions on cancer patients. Indeed, several randomized controlled trials explored this issue [17,18,19,20]. To the best of our knowledge, there is no relevant systematic review that has summarized this evidence. Therefore, the purpose of our systematic review was to examine the effects of any physical activity/exercise intervention combined with any diet/nutrition intervention on any biological/biochemical index, quality of life (QoL), and depression in breast, lung, colon and rectum, prostate, stomach, and liver cancer patients and/or cancer survivors.

## 2. Materials and Methods

A systematic review and meta-analysis were undertaken, using the Preferred Reporting Items for Systematic Reviews and Meta-analyses (PRISMA) guidelines [21] and the Cochrane Handbook [22]. We registered a systematic review in the International Prospective Register of Systematic Reviews (PROSPERO) database; registration number: CRD42023481429 [23].

### 2.1. Searching and Selection Processes

PubMed, EMBASE, and SportDiscus were explored from their inception to November 2023. We applied no limitations in the search for the date, language of publication, and study design. The search algorithm for PubMed is shown in Supplement S1 (pages 1–2). The co-authors shaped the keyword algorithm, and the search was completed by PCD and tested by GSM. Seven teams of co-authors were created to execute the selection procedure. Each team assessed an equal number of retrieved publications according to the eligibility criteria. The results of the selection procedure were tested by PCD, and any disagreements were resolved by a referee (GSM). Finally, PCD screened the reference lists of eligible publications to identify any additional eligible publications.

### 2.2. Inclusion and Exclusion Criteria

The eligible studies included the following:

1. Population: Adult human (>18 years) breast, lung, colon and rectum, prostate, stomach, and liver cancer patients, and/or cancer survivors. Patients at any cancer stage, with any comorbidity, at any body mass index (BMI), or under any pharmacological treatment were included.
2. Intervention: Any physical activity/exercise intervention combined with any diet/nutrition intervention, as well as physical and/or remote counseling, behavioral models, education as a combined physical activity/exercise, and diet/nutrition intervention, as long as this intervention was specific and measurable. Interventions took place for at least two weeks.
3. Comparator: As a control condition, we included randomized controlled trials (RCT) with a control group (i.e., usual care) and RCT with a cross-over design control condition.
4. Outcome: Measurement of biological/biochemical indices, QoL, and depression.
5. Study design: RCTs with parallel or cross-over design.
6. We rejected studies that involved animals, reviews, letters, congress papers, magazines, and gray literature.

### 2.3. Risk of Bias Assessment

Τhe Risk of Bias 2 (RoB2) Cochrane library tool was incorporated to assess the eligible RCTs [24]. Each team of co-authors evaluated an equal number of eligible papers. PCD coordinated the process, and GSM acted as a referee in the case of conflicts.

### 2.4. Data Extraction

The following data were extracted: (a) first author name and year of publication, (b) participants’ anthropometric characteristics (i.e., age, gender, and BMI), (c) cancer type and cancer stage, (d) physical activity/exercise interventions/measurements, (e) diet/nutrition interventions/measurements, and (f) outcomes. The study characteristics are displayed in Supplement S1 (Table S1, pages 3–17). An initial data extraction calibration was performed to determine the type of data and format that should be extracted. Each team of co-authors extracted data from an equal number of eligible publications. PCD coordinated the data extraction procedure, and GSM acted as a referee in the case of conflicts.

### 2.5. Data Synthesis

We incorporated a narrative data synthesis for eligible studies that offered no data for meta-analyses. To conduct our meta-analyses, we used a random-effect model due to the heterogeneity of the eligible studies in characteristics, such as populations, type of cancer, physical activity/exercise, and diet/nutrition interventions and duration. We used the RevMan 5.4.1, 2020 software (The Cochrane Collaboration, London, UK) [25] to perform the meta-analyses. An inverse variance continuous method was incorporated to test the mean differences (MD) between a combined intervention of physical activity/exercise and diet/nutrition and a control group/condition. A standardized mean difference (SMD) method was used for different units of the variables included in the meta-analysis [22]. The following variables were used in our meta-analyses: BMI, body weight (kg), fat mass (kg), fat-free mass (kg), insulin levels, homeostatic model assessment for insulin resistance (HOMA-IR), glucose, C-reactive protein, high-density lipoprotein (HDL), low-density lipoprotein (LDL), triglycerides, bone mineral density, components of QoL (physical, mental, social, and bodily pain), and depression. Standard errors were converted into standard deviations (SD) using the equation: $SD=standard\;error*\sqrt{n}$ [22]. Similarly, in the case of medians and if the 1st–3rd quartiles were provided, we calculated the mean and SD using the following equation: *mean* = (*q*1 + *m* + *q*3)/3; SD = (*q*3 − *q*1)/1.35 [22,26]. Overall, from the 38 eligible RCTs, 30 offered data for a meta-analysis [17,18,19,20,27,28,29,30,31,32,33,34,35,36,37,38,39,40,41,42,43,44,45,46,47,48,49,50,51,52]. For all meta-analyses, we created forest plots, but we produced funnel plots when a meta-analysis included >10 studies [22].

### 2.6. Quality of Evidence

We evaluated the quality of evidence for each meta-analysis using the Grading of Recommendations Assessment, Development, and Evaluation (GRADE) analysis [22,53].

## 3. Results

### 3.1. Searching and Selection Processes Results

The search procedure yielded 4337 publications. We removed 306 duplicate publications, and 848 were ineligible by screening their titles. The remaining 3183 publications were screened for eligibility (titles, abstract, and full texts), and 2037 of them were excluded, given that they were reviews, editorials, case reports, consensus papers, and conference proceedings. We also excluded 1113 publications that did not fulfill the eligibility criteria. Thus, 33 publications were included as eligible. We then screened the reference lists of these 33 publications (overall 1594 citations) and we identified five more eligible publications that did not appear in the initial search procedure. In total, 38 publications were finally included as eligible for our systematic review. A PRISMA flow diagram can be found in Supplement S1 (Figure S1, page 18).

### 3.2. Risk of Bias Assessment Results

**Figure 1:**
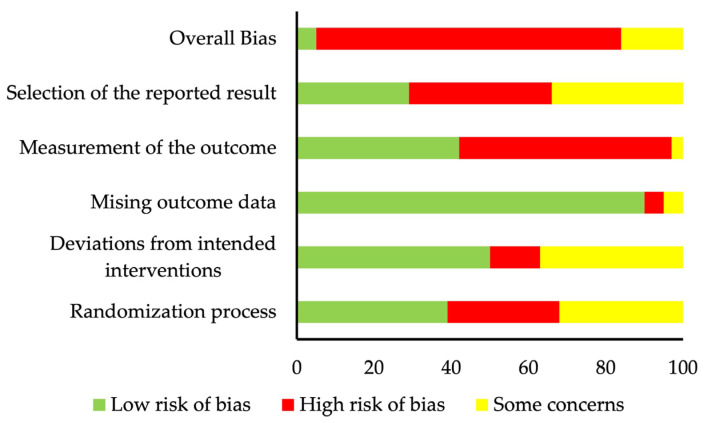
Summary of risk of bias assessment.

In total, 30 (79%) out of 38 eligible studies displayed an overall high risk of bias, mainly due to a high risk of bias in the “measurement of the outcome” (55% of the studies) and the “selection of the reported results” (37% of the studies). Two (5%) eligible studies [27,31] showed an overall low risk, and six (16%) eligible studies [18,30,38,45,48,54] overall displayed some concerns of bias. The detailed risk of bias assessment results can be found in Table S2 in Supplement S1 (pages 19–20), and a summary of the risk of bias can be found in Figure 1.

### 3.3. Narrative Data Synthesis Results

Eight eligible studies [54,55,56,57,58,59,60,61] did not offer data for a meta-analysis; therefore, a narrative data synthesis approach was used. The results of this synthesis can be found in Table 1.

### 3.4. Meta-Analysis Outcomes

#### 3.4.1. Biological/Biochemical Indices

**Figure 2:**
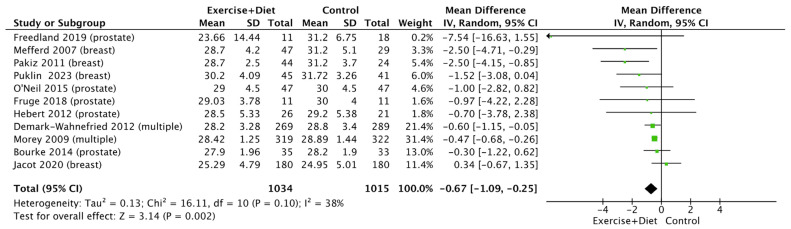
Forest plot of the effects of physical activity/exercise and diet/nutrition intervention on body mass index. SD: standard deviation; CI: confidence interval.

We found that the intervention reduced BMI [(MD = −0.67, confidence interval (CI) = (−1.09)–(−0.25), Z = 3.14, I^2^ = 38%, and *p* = 0.002; Figure 2, Figure S2 in Supplement S1, page 20]. Subgroup analysis revealed no differences between cancer types (*p* > 0.05; Figure S3 in Supplement S1, page 20).

**Figure 3:**
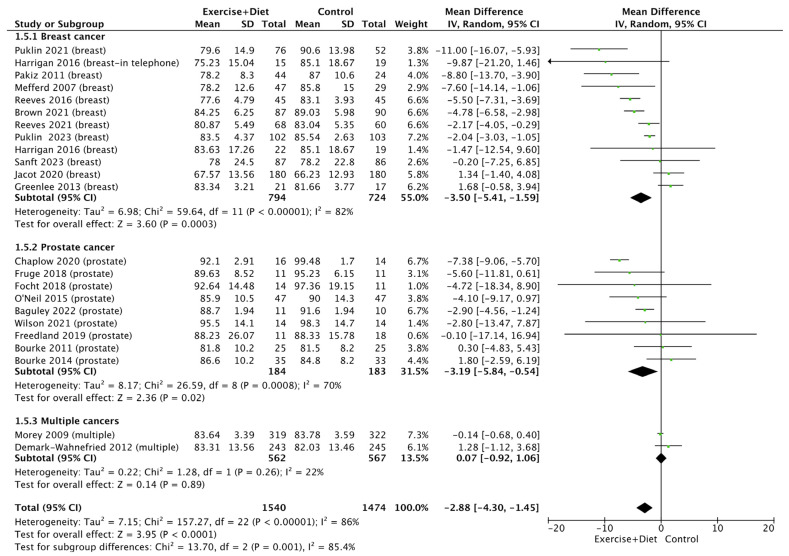
Forest plot of the effects of physical activity/exercise and diet/nutrition intervention on body weight. SD: standard deviation; CI: confidence interval.

We also found that the intervention decreased body weight [(MD = −2.88, CI = (−4.30)–(−1.45), Z = 3.95, I^2^ = 86%, and *p* < 0.0001; Figure 3, Figure S4 in Supplement S1, page 21]. Subgroup analysis (*p* = 0.001) revealed that the intervention was more effective in breast cancer (MD = −3.50) than in prostate cancer (MD = −3.19) and in patients with multiple cancers (MD = 0.07); Figure 3.

**Figure 4:**
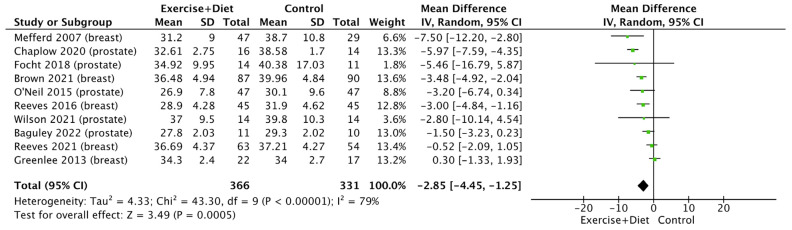
Forest plot of the effects of physical activity/exercise and diet/nutrition intervention on fat mass. SD: standard deviation; CI: confidence interval.

The fat mass of cancer patients and survivors was also reduced due to the intervention [(MD = −2.85, CI = (−4.45)–(−1.25), Z = 3.49, I^2^ = 79%, and *p* = 0.0005; Figure 4, Figure S5 in Supplement S1, page 21]. Subgroup analysis showed no differences between cancer types (Figure S6 in Supplement S1, page 22).

**Figure 5:**
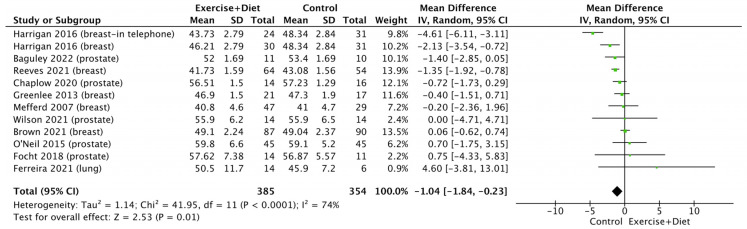
Forest plot of the effects of physical activity/exercise and diet/nutrition intervention on fat-free mass. SD: standard deviation; CI: confidence interval.

A meta-analysis of the effects of the intervention on fat-free mass showed that this was reduced in the control group [(MD = −1.04, CI = (−1.84)–(−0.23), Z = 2.53, I^2^ = 74%, and *p* = 0.01; Figure 5, Figure S7 in Supplement S1, page 22], with no differences between cancer types (*p* > 0.05; Figure S8 in Supplement S1, page 23).

**Figure 6:**
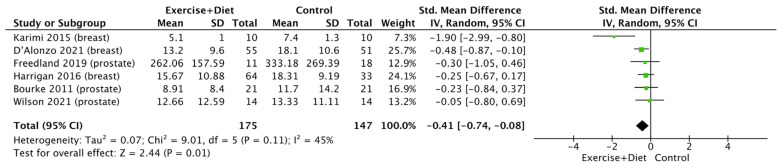
Forest plot of the effects of physical activity/exercise and diet/nutrition intervention on insulin levels. SD: standard deviation; CI: confidence interval.

Six studies offered data for the meta-analysis of insulin levels. This revealed that the intervention decreased insulin levels compared to the control group [(SMD = −0.41, CI = (−0.74)–(−0.08), Z = 2.44, I^2^ = 45%, *p* = 0.01; Figure 6], with no differences between cancer types (*p* > 0.05; Figure S9 in Supplement S1, page 23).

**Figure 7:**
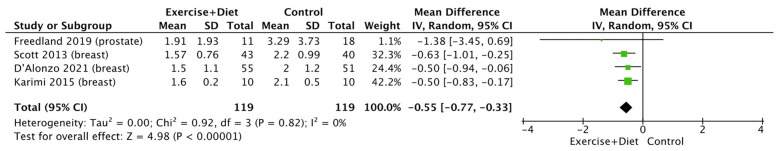
Forest plot of the effects of physical activity/exercise and diet/nutrition intervention on homeostatic assessment model for insulin resistance. SD: standard deviation; CI: confidence interval.

In line with insulin levels, a meta-analysis of HOMA-IR showed that the intervention reduced insulin levels [(SMD = −0.55, CI = (−0.77)–(−0.33), Z = 4.98, I^2^ = 0%, and *p* < 0.00001; Figure 7], whereas there was no effect on glucose levels (*p* > 0.05; Figure S10 in Supplement S1, page 23).

**Figure 8:**
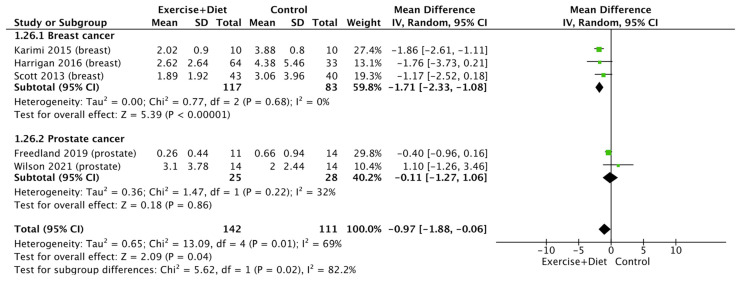
Forest plot of the effects of physical activity/exercise and diet/nutrition intervention on C-reactive protein. SD: standard deviation; CI: confidence interval.

Regarding the chronic inflammation indices of cancer patients and survivors, a meta-analysis of C-reactive protein showed that the intervention reduced this [(MD = −0.97, CI = (−1.88)–(−0.06), Z = 2.09, I^2^ = 69%, and *p* = 0.01; Figure 8], whereas a subgroup analysis revealed that this occurs only in patients and survivors with breast cancer but not in those with prostate cancer (*p* = 0.02 and I^2^ = 82.2%; Figure 8).

**Figure 9:**
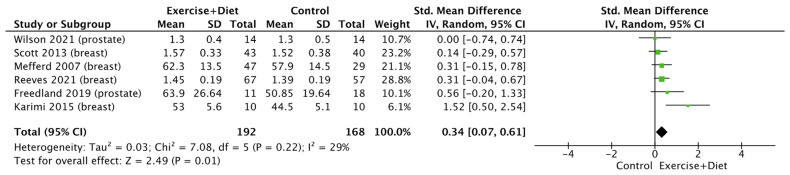
Forest plot of the effects of physical activity/exercise and diet/nutrition intervention on high-density lipoprotein. SD: standard deviation; CI: confidence interval.

**Figure 10:**
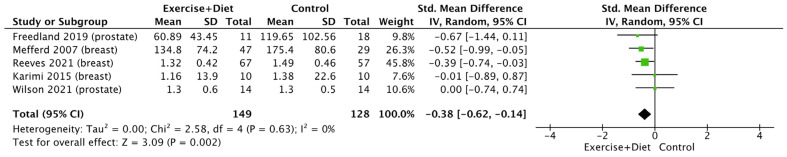
Forest plot of the effects of physical activity/exercise and diet/nutrition intervention on triglycerides. SD: standard deviation; CI: confidence interval.

Regarding the lipidemic profile of the participants, HDL increased due to the intervention (SMD = 0.34, CI = 0.07–0.61, Z = 2.49, I^2^ = 29%, and *p* = 0.01; Figure 9), whereas there were no differences between cancer types (*p* > 0.05; Figure S11 in Supplement S1, page 24). Additionally, the intervention had no effect on LDL (*p* > 0.05; Figure S12 in Supplement S1, page 24), whereas the intervention decreased triglycerides [(SMD = −0.38, CI = (−0.62)–(−0.14), Z = 3.09, I^2^ = 0%, and *p* = 0.002; Figure 10], with no differences between cancer types (*p* > 0.05; Figure S13 in Supplement S1, page 24). Finally, no effect of the intervention was found in bone mineral density (*p* > 0.05; Figure S14 in Supplement S1, page 25).

#### 3.4.2. Quality of Life Indices

**Figure 11:**
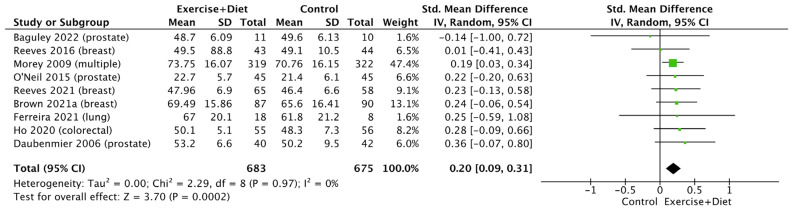
Forest plot of the effects of physical activity/exercise and diet/nutrition intervention on quality of life physical component summary score. SD: standard deviation; CI: confidence interval.

**Figure 12:**
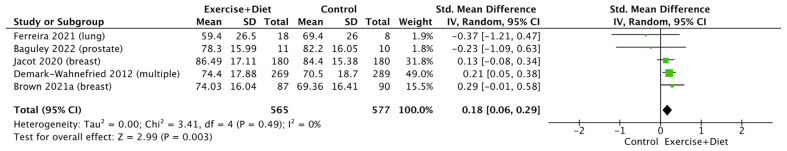
Forest plot of the effects of physical activity/exercise and diet/nutrition intervention on quality of life physical functioning. SD: standard deviation; CI: confidence interval.

Meta-analyses on QoL indices showed no effect of the intervention on the mental health summary score (*p* > 0.05; Figure S15 in Supplement S1, page 25), social functioning (*p* > 0.05; Figure S16 in Supplement S1, page 25), and bodily pain (*p* > 0.05; Figure S17 in Supplement S1, page 25). On the other hand, the intervention showed an increase in the physical component summary score (SMD = 0.20, CI = 0.09–0.31, Z = 3.70, I^2^ = 0%, and *p* = 0.0002; Figure 11), with no differences between cancer types (*p* > 0.05; Figure S18 in Supplement S1, page 26). Similarly, the intervention increased QoL physical functioning (SMD = 0.18, CI = 0.06–0.29, Z = 2.99, I^2^ = 0%, and *p* = 0.003; Figure 12), with no differences between cancer types (*p* > 0.05; Figure S19 in Supplement S1, page 26).

**Figure 13:**
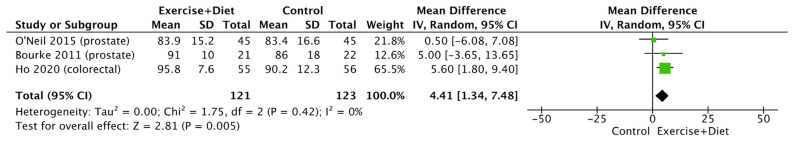
Forest plot of the effects of physical activity/exercise and diet/nutrition intervention on the general functional assessment of cancer therapy. SD: standard deviation; CI: confidence interval.

**Figure 14:**
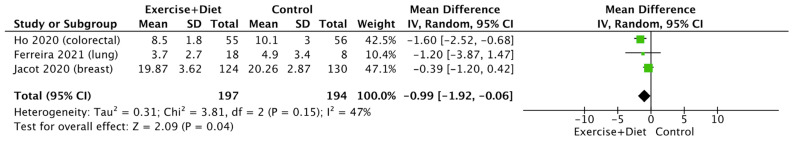
Forest plot of the effects of physical activity/exercise and diet/nutrition intervention on depression. SD: standard deviation; CI: confidence interval.

Finally, the meta-analyses revealed that the intervention increased the general functional assessment of cancer therapy (FACT-general) (MD = 4.41, CI = 1.34–7.48, Z = 2.81, I^2^ = 0%, and *p* = 0.005; Figure 13), as well as decreased depression (MD = −0.99, CI = (−1.92)–(−0.06), Z = 2.09, I^2^ = 47%, and *p* = 0.04; Figure 14) in cancer patients and survivors.

### 3.5. Quality of Evidence Results

The GRADE analysis revealed 2 out of 13 meta-analyses as low-quality evidence, while 11 meta-analyses were rated as moderate quality, mainly because of the high risk of bias that the eligible studies displayed. The outcomes of the GRADE analysis can be found in Table 2, and the detailed GRADE analysis for each meta-analysis can be found in Supplement S2.

## 4. Discussion

### 4.1. Summary of Main Findings

The current systematic review and meta-analysis revealed moderate-quality evidence that combined intervention of physical activity/exercise and diet/nutrition reduced BMI, insulin, HOMA-IR, C-reactive protein, triglycerides, and depression in the intervention group, while it reduced fat-free mass in the control group. The meta-analysis also revealed low-quality evidence that the intervention reduced body weight and fat mass, while moderate-quality evidence showed that it increased HDL, QoL physical component summary score, QoL physical functioning, and FACT general.

The narrative data synthesis showed that combined intervention of physical activity/exercise and diet/nutrition had no effect on sex steroid hormones and breast cancer-related lymphedema. On the other hand, the intervention reduced oxidative stress and improved psychological, physiological, and behavioral outcomes, while it had an anti-dysmetabolism effect, leading to telomere lengthening, delayed conventional treatment, and modified dietary habits.

### 4.2. Completeness and Applicability of Evidence

We found an adequate number of studies with an adequate sample size (n > 5000) to test whether the combined intervention of physical activity/exercise and diet/nutrition can affect the body weight and body composition of cancer patients and survivors. Our results showed that the intervention reduced BMI, body weight, and fat mass by 0.67 kg/m^2^, 2.88 kg, and 2.85 kg, respectively, within a period of 3–12 months. Considering that there is a positive association between BMI, body weight, and fat mass [62], our findings are particularly important given that excessive body weight and adiposity can enhance cancer development [63]. Also, a reduction in fat mass is associated with a generally favorable metabolic profile (e.g., reduced circulating insulin levels) [64] and an inflammatory profile [65]. Indeed, excessive adipose tissue is associated with higher levels of insulin, which may enhance the proliferation of cancer cells [64]. Our meta-analysis showed that a combined intervention of physical activity/exercise and diet/nutrition reduced the insulin and HOMA-IR levels. A potential explanation is that physical activity/exercise can increase insulin sensitivity through increased expression of Glucose transporter-4 in the plasma membrane of skeletal muscle cells [66], while a reduction in sweet drinks, sweets, and consumption of low glycemic index food, as well as diets rich in fibers, may improve insulin sensitivity [67]. Regarding inflammation, we found that a combined intervention of physical activity/exercise and diet/nutrition reduced C-reactive protein, indicating a reduction in chronic inflammation, and thus, a reduction in carcinogenesis risk [63]. Collectively, a combined intervention of physical activity/exercise and diet/nutrition has the potential to reduce cancer development, which is particularly important in cancer survivors.

A previous systematic review showed that a combination of exercise and weight loss through energy-restricted diets can effectively reduce fat mass in overweight and obese individuals without posing a risk for sarcopenia [68]. In our meta-analysis, we found moderate-quality evidence that the control group of cancer patients and survivors reduced their fat-free mass during the period of the intervention. This is not a favorable effect, given that fat-free mass, particularly muscle mass, may produce a number of myokines with anti-inflammatory effects and increased insulin sensitivity, which is consequently associated with a reduced risk of cancer development [65]. A possible explanation for our finding is that cancer mortality is associated with decreased lean mass [69], indicating that cancer patients display relatively low levels of lean mass, including muscle mass. The extent to which we apply a combined intervention of physical activity/exercise and diet/nutrition to cancer patients and survivors should be approached with caution to avoid muscle mass reduction. One suggestion is to use a combination of resistance exercise and diets rich in protein, known to increase muscle mass [70].

We also detected moderate-quality evidence that a combined intervention of physical activity/exercise and diet/nutrition reduced triglycerides and increased HDL levels. Physical activity/exercise is associated with decreased levels of triglycerides and increased levels of HDL [71], whereas several nutrient consumption and/or diets have decreased triglycerides and increased HDL [72]. This beneficial effect may reduce cancer risk due to the involvement of triglycerides in the pathogenesis of several cancers, including lung, prostate, and rectal cancer [73]. We also detected moderate-quality evidence that a combined intervention of physical activity/exercise and diet/nutrition reduced depression in cancer patients and survivors. Depression results in higher rates of mortality among cancer patients [15,74] and poor QoL [15]. Physical activity/exercise reduces depression levels, while high intensities of physical activity/exercise cause euphoria due to increased production of β-endorphin in the brain, indicating decreased levels of depression [16]. Furthermore, a 12-week aerobic exercise program decreased depression levels [75], whereas a Cochrane systematic review showed that yoga exercise can reduce short-term symptoms of depression, anxiety, and fatigue in breast cancer patients [76]. Also, certain fatty acids (i.e., n-3 long-chain polyunsaturated, omega-3) consumption may improve depressive symptoms [77,78]. Depression is closely associated with poor QoL [15]. We found moderate-quality evidence that a combined intervention of physical activity/exercise and diet/nutrition improved both general physical functioning and functional assessment of cancer therapy in cancer patients and survivors. This outcome shows the already reported beneficial effect of physical activity/exercise on QoL in clinical populations [79], whereas the effect of dietary interventions on QoL is not well documented [80].

The narrative data synthesis offered limited data to answer our research question. There was an opposite outcome between the two studies [56,58] regarding the effects of a combined intervention of physical activity/exercise and diet/nutrition on telomere length. A previous systematic review showed that higher adherence to the Mediterranean diet is related to longer telomere length [81], whereas another systematic review showed that various dietary interventions are not associated with telomere length [82]. This evidence may partly explain the opposite outcome we observed in our systematic review, given that the diet interventions in the included studies were not entirely related to the Mediterranean diet (Table 1). Regarding exercise, the opposite outcome may be explained by the fact that the study that found no effect of the combined intervention on telomere length [56] used resistance along with aerobic exercise intervention. A previous systematic review showed that only aerobic exercise may improve telomere length [83], which was used in our included study [58] and showed a positive effect on telomere length. The rest of the narrative data synthesis outcomes are general and disparate.

The overall applicability of the evidence is moderate, based on the quality of the available evidence in the current systematic review and meta-analysis. However, given the heterogeneity of the interventions included in our meta-analysis (i.e., different physical activity/exercise types and modes and various diets and supplements), the outcomes of our systematic review should be treated with specific analysis. For instance, considering the specific characteristics of the populations and interventions involved in our systematic review, to apply interventions in practice.

### 4.3. Strengths and Limitations

The strengths of our systematic review include the development of a search algorithm using standardized indexing terms, which may capture papers that use another keyword to describe the same term [22]. Screening of eligible studies, risk of bias assessment, and data extraction were performed by two independent investigators. We accepted studies in any language and from any time of publication. Finally, we evaluated the quality of the meta-analyses using the GRADE analysis, which added value to their interpretation.

Limitations of our systematic review include the large heterogeneity of the populations, especially in the type of measurements, physical activity/exercise, and diet/nutrition schemes, which may have affected the interpretation of the results. Also, it was not possible to consider potential medicine schemes that were received by the participants due to missing information in the eligible publications. This may have affected the results of the eligible studies and, consequently, our systematic review results.

### 4.4. Important Deviations from the Published Protocol

We report no significant deviations from the published protocol [23].

## 5. Conclusions

We conclude that a combined intervention of physical activity/exercise and diet/nutrition may decrease body weight, fat mass, insulin levels, and inflammation and improve lipidemic profiles in cancer patients and survivors. We also conclude that a combined intervention of physical activity/exercise and diet/nutrition may improve the physical component of QoL, as well as depression levels, in cancer patients and survivors. These outcomes indicate a lower risk for carcinogenesis; however, their applicability depends on the heterogeneity of the population and interventions, as well as the potential medical treatment of cancer patients and survivors.

## Figures and Tables

**Table 1:** Narrative data synthesis results. Green color represents a positive effect of the intervention; orange color represents no effect of the intervention; QoL = quality of life; BMI = body mass index; WCRF = World Cancer Research Fund.

Study	Aim	Type of Exercise/Physical Activity and Diet/Nutrition	Outcome
Brown 2022 (Breast cancer) [55]	To examine the effects of the intervention (physical activity and diet) on sex steroid hormones.	Duration: 52 weeks. Exercise Type: Resistance + aerobic. Exercise Content: 9 exercises twice weekly, 2–3 sets, weight permitted 10 repetitions. Moderate-intensity aerobic exercise, 180 min 3–6 days/week. Diet Type: Hypocaloric diet 10% loss of body weight. Diet Content: 7 daily servings of fruits and vegetables; behavioral techniques to prepare food.	No effect of the intervention on sex steroid hormones.
Schmitz 2019 (Breast cancer) [59]	To examine the effects of the intervention (physical activity and diet) on breast cancer-related lymphedema.	Duration: 52 weeks. Exercise Type: Walking + resistance exercises Exercise Content: Walking goals/week were 90 min for weeks 1–3, 120 min for week 4, 150 min for weeks 5–6, and 180 min thereafter. Intensity was limited by 0.45–0.90 kg per 2 weeks, up to 9.45 kg, twice/session during weeks 1–6 and 3 times/session thereafter. Diet Type: Weight loss. Diet Content: Weeks 1–20: 7 servings of fruits and vegetables per day. Weeks 21–24 lessons for preparing food. Weeks 25–52, monthly meetings for weigh-ins and weight maintenance instructions, to achieve 10% weight reduction in comparison to baseline.	The intervention did not improve breast cancer-related lymphedema.
Brown 2023 (Breast cancer) [56]	To examine the effects of the intervention (physical activity and diet) on oxidative stress and telomere length.	Duration: 52 weeks Exercise Type: Resistance + aerobic. Exercise Content: 9 exercises twice weekly, 2–3 sets, weight permitted 10 repetitions. Moderate-intensity aerobic exercise, 180 min 3–6 days/week. Diet Type: Hypocaloric diet 10% loss of body weight. Diet Content: 7 portions of fruits and vegetables per day; behavioral techniques to prepare food.	The intervention was associated with reduced oxidative stress. No effect of the intervention on telomere length.
Carayol 2019 (Breast cancer) [57]	To examine the effects of the intervention (physical activity and diet) on cancer-related fatigue, QoL, anxiety, depression, BMI, and body composition.	Duration: 26 weeks. Exercise Type: Resistance + aerobic. Exercise Content: 1 resistance session/week, 2–5 sets, 6–12 repetitions. Two moderate-intensity 30–45 min aerobic sessions/week, 50–75% maximum heart rate. Diet Type: Balanced dietary intake. Diet Content: Diet prepared according to WCRF.	The intervention had positive changes in terms of psychological, physiological, and behavioral outcomes.
Karimi 2013 (Breast cancer) [54]	To examine the effects of the intervention (physical activity and diet) on adiponectin and oxidative stress.	Duration: 6 weeks. Exercise Type: Water-based exercise. Exercise Content: 50–75% of heart rate reserve, in a pool, 4 times/week. Diet Type: Oral ginger supplement. Diet Content: Ginger rhizome powder (750 mg) in 250 mL of water, 4 times/day, during all main meals and in the afternoon.	The intervention had an antioxidant and anti-dysmetabolism effect.
Sanft 2018 (Breast cancer) [58]	To examine the effects of the intervention (physical activity and diet) on telomere length.	Duration: 6 months. Exercise Type: Home-based exercise (walking). Exercise Content: 150 min/week of moderate-intensity activity and 10.000 steps/day. Diet Type: Reduced caloric intake. Diet Content: Reduction of 1200–2000 kcal/day, based on baseline weight, and decreasing fat to <25% of total energy intake.	The intervention led to telomere lengthening.
Frattaroli 2008 (Prostate cancer) [60]	To examine the effects of the intervention (physical activity and diet) on prostate-specific antigen.	Duration: 2 years. Exercise Type: Moderate aerobic exercise. Exercise Content: Walking 30 min/day, 6 days/week. Diet Type: Vegan diet. Diet Content: Fruits, vegetables, whole grains, legumes, and soy products, low in carbohydrates, and 10% of calorie intake from fat.	The intervention allowed active surveillance to delay conventional treatment.
Lee 2018 (Colorectal cancer) [61]	To examine the effects of the intervention (physical activity and diet) on consumption of red and processed meat.	Duration: 12 months. Exercise Type: Moderate to vigorous physical activity. Exercise Content: 60 min of moderate-vigorous physical activity, 5 days/week. Diet Type: Consultation using interviews and phone calls. Diet Content: High dietary fiber, low red and processed meat, and refined grain.	The intervention showed potential for the cancer survivors to modify their dietary habits.

**Table 2:** GRADE analysis outcomes: CI: confidence interval; BMI: body mass index; MD: mean difference; SMD: standardized mean difference; HOMA-IR: homeostatic assessment mode for insulin resistance; HDL: high-density lipoprotein; FACT: functional assessment of cancer therapy.

Outcomes	No of Participants (Studies/Entries)	Quality of the Evidence (GRADE)	Relative Effect (95% CI)
Exercise+Diet vs. Control BMI	2049 (11 studies/entries)	Moderate ⨁⨁⨁◯ due risk of bias	MD: −0.67, CI: −1.09, −0.25
Exercise+Diet vs. Control Body weight	3014 (23 studies/entries)	Low ⨁⨁◯◯ due to risk of bias and inconsistency of results	MD: −2.88, CI: −4.30, −1.25
Exercise+Diet vs. Control Fat mass	697 (10 studies/entries)	Low ⨁⨁◯◯ due to risk of bias and inconsistency of results	MD: −2.85, CI: −4.45, −1.25
Exercise+Diet vs. Control Fat-Free mmass	739 (12 studies/entries)	Moderate ⨁⨁⨁◯ due risk of bias	MD: −1.04, CI: −1.84, −0.23
Exercise+Diet vs. Control Insulin	322 (6 studies/entries)	Moderate ⨁⨁⨁◯ due risk of bias	SMD: −0.41, CI: −0.74, −0.08
Exercise+Diet vs. Control HOMA-IR	238 (4 studies/entries)	Moderate ⨁⨁⨁◯ due risk of bias	MD: −0.55, CI: −0.77, −0.33
Exercise+Diet vs. Control C-reactive protein	253 (5 studies/entries)	Moderate ⨁⨁⨁◯ due risk of bias	MD: −0.97, CI: −1.88, −0.06
Exercise+Diet vs. Control HDL	360 (6 studies/entries)	Moderate ⨁⨁⨁◯ due risk of bias	SMD: 0.34, CI: 0.07, 0.61
Exercise+Diet vs. Control Triglycerides	297 (5 studies/entries)	Moderate ⨁⨁⨁◯ due risk of bias	SMD: −0.38, CI: −0.62, −0.14
Exercise+Diet vs. Control QoL physical component summary score	1358 (9 studies/entries)	Moderate ⨁⨁⨁◯ due risk of bias	SMD: 0.20, CI: 0.09, 0.31
Exercise+Diet vs. Control QoL physical functioning	1142 (5 studies/entries)	Moderate ⨁⨁⨁◯ due risk of bias	SMD: 0.18, CI: 0.06, 0.29
Exercise+Diet vs. Control FACT general	244 (3 studies/entries)	Moderate ⨁⨁⨁◯ due risk of bias	MD: 4.41, CI: 1.34, 7.48
Exercise+Diet vs. Control Depression	391 (3 studies/entries)	Moderate ⨁⨁⨁◯ due risk of bias	MD: −0.99, CI: −1.92, −0.06

## Data Availability

Extracted data used in the meta-analyses are available upon reasonable request.

## Supplementary Materials

The following supporting information can be downloaded at: https://www.mdpi.com/article/10.3390/nu16111749/s1, Figure S1. PRISMA flow diagram; Figure S2. Funnel plot on the effects of diet/nutrition and physical activity/exercise intervention on body mass index; Figure S3. Subgroup analysis of the effects of diet/nutrition and physical activity/exercise intervention on body mass index; Figure S4. Funnel plot on the effects of diet/nutrition and physical activity/exercise intervention on body weight; Figure S5. Funnel plot on the effects of diet/nutrition and physical activity/exercise intervention on fat mass; Figure S6. Subgroup analysis of the effects of diet/nutrition and physical activity/exercise intervention on fat mass; Figure S7. Funnel plot of the effects of diet/nutrition and physical activity/exercise intervention on fat free mass; Figure S8. Subgroup analysis of the effects of diet/nutrition and physical activity/exercise intervention on fat free mass; Figure S9. Subgroup analysis of the effects of diet/nutrition and physical activity/exercise intervention on insulin levels; Figure S10. Subgroup analysis of the effects of diet/nutrition and physical activity/exercise intervention on glucose levels; Figure S11. Subgroup analysis of the effects of diet/nutrition and physical activity/exercise intervention on HDL; Figure S12. Forest plot of the effects of diet/nutrition and physical activity/exercise intervention on LDL; Figure S13. Subgroup analysis of the effects of diet/nutrition and physical activity/exercise intervention on triglycerides; Figure S14. Forest plot of the effects of diet/nutrition and physical activity/exercise intervention on bone mineral density; Figure S15. Forest plot of the effects of diet/nutrition and physical activity/exercise intervention on QoL mental health summary score; Figure S16. Forest plot of the effects of diet/nutrition and physical activity/exercise intervention on QoL social functioning; Figure S17. Forest plot of the effects of diet/nutrition and physical activity/exercise intervention on QoL bodily pain; Figure S18. Subgroup analysis of the effects of diet/nutrition and physical activity/exercise intervention on QoL physical component summary score; Figure S19. Subgroup analysis of the effects of diet/nutrition and physical activity/exercise intervention on QoL physical functioning; Table S1. Characteristics of eligible studies. BMI: body mass index; QoL: quality of life; WCRF: world cancer research fund; HIIT: high intensity interval training; NR: not reported; HR: heart rate; RM: repetition maximum; CI: confidence interval; IQR: interquartile range; CDC: centre for disease control; ACSM: American college of sport medicine; HOMA-IR: homeostatic model assessment-insulin resistance. Table S2. Risk of bias assessment results.

## Appendix A

List of students who meet the criteria for co-authors (in alphabetical order)

	**Full Name**	**Email**
1	Apostolos Kamnitsas	akamnitsas@uth.gr
2	Aristoula Tzalidi	atzalidi@uth.gr
3	Aikaterini Pipertzi	apipertzi@uth.gr
4	Aikaterini Makedoniti	amakedoniti@uth.gr
5	Aikaterini Makri	aikmakri@uth.gr
6	Asteria Skliri	askliri@uth.gr
7	Asteria Sideri Tsiami	asideri-tsami@uth.gr
8	Anastasia Riyinou	ariginou@uth.gr
9	Alkmini Rouchota	arouchota@uth.gr
10	Anastasia Kraniotaki	akraniotaki@uth.gr
11	Aggeliki Chalvatzi	anchalvantzi@uth.gr
12	Anastasia Kanaridou	akanaridou@uth.gr
13	Ariandi Marinou	armarinou@uth.gr
14	Angelos Chaniotakis	achaniotakis@uth.gr
15	Alexandros Foteinos	afoteinos@uth.gr
16	Apostolos Aggelos Patsatzis	apatsatzis@uth.gr
17	Argiro Diotima Tiktopoulou	atiktopoulou@uth.gr
18	Chariklia Fygka	cfygka@uth.gr
19	Chariklia Petropoulou	cpetropoulou@uth.gr
20	Chrisanthi Kyriazi Theodori	chkyriazi@uth.gr
21	Dimitris Grigoriou	dimgrigoriou@uth.gr
22	Eirini Mitou	emitou@uth.gr
23	Eirini Mpalampani	ebalampani@uth.gr
24	Eirini Pavlou	eirpavlou@uth.gr
25	Eugenia Anthi	eanthi@uth.gr
26	Evdokia Chatzimanoli	evchatzimanoli@uth.gr
27	Eleni Sofia Kapsokavadi	ekapsokavadi@uth.gr
28	Euaggelia Vordoni	evordoni@uth.gr
29	Eleni Papadima	elpapadima@uth.gr
30	Elisavet Pappa	elipappa@uth.gr
31	Ermioni Dimopoulou	erdimopoulou@uth.gr
32	Georgia Fafaliou	gfafaliou@uth.gr
33	Georgios Kalogiannakis	gkalogiannakis@uth.gr
34	Ioanna Apostolou	ioapostolou@uth.gr
35	Ioannis Manolarakis	imanolarakis@uth.gr
36	Konstantina Stamou	kstamou@uth.gr
37	Konstantina Michailidou	konmichailidou@uth.gr
38	Maria Gerostergiou	mgerostergiou@uth.gr
39	Maria Liveri	mliveri@uth.gr
40	Maria Mpoufikou	maboufikou@uth.gr
41	Maria Paschalidou	mapaschalidou@uth.gr
42	Marianthi Aristea Vasilopoulou	mvasilopoul@uth.gr
43	Marianthi Koropouli	vkotopouli@uth.gr
44	Nikolaos Gerasimos Efthimiadis	niefthymiadis@uth.gr
45	Niki Stathi	nstathi@uth.gr
46	Niki Ioanna Karanikola	nkaranikola@uth.gr
47	Nektaria Dimitra Kotsiari	nkotsiari@uth.gr
48	Paoulina Maria Pietchoux	ppietsouch@uth.gr
49	Paraskevi Mpourda	pbourda@uth.gr
50	Petros Stefanidis	pstefanidis@uth.gr
51	Sevastiani Voulgaraki	svoulgaraki@uth.gr
52	Vasiliki Kotopouli	vkotopouli@uth.gr
53	Vasiliki Paraskevi Voutiritsa	voutyritsa@uth.gr
54	Vasiliki Alexaki	valexaki@uth.gr
55	Zoi Nona	znona@uth.gr
56	Zinovia Βermperi	zbermperi@uth.gr
