# Supplementary material for: Combined Effects of Physical Activity and Diet on Cancer Patients: A Systematic Review and Meta-Analysis
Source: Nutrients. 2024 Jun 2;16(11):1749. doi: 10.3390/nu16111749 (PMC11175154; doi:10.3390/nu16111749)

SUPPLEMENT S1

Combined effects of physical activity and diet on cancer patients. A systematic review and meta-analysis

Petros C. Dinas[1,2,*], on behalf of the students of module 5104 (Introduction to Systematic Reviews)[1,+,] Marianthi Karaventza[1], Christina Liakou[3], Kalliopi Georgakouli,[1] Dimitrios Bogdanos[4], and George S. Metsios[1]

[1] Department of Nutrition and Dietetics, School of Physical Education, Sport Science and Dietetics, University of Thessaly, 42130 Trikala, Greece; g.metsios@uth.gr (G.S.M.), markaraventza@gmail.com (MK)
[2] FAME Laboratory, School of Physical Education, Sport Science and Dietetics, University of Thessaly, 42131 Trikala, Greece
[3] School of Physical Education, Sport Science and Dietetics, University of Thessaly, 42131 Trikala, Greece; cliakou@uth.gr
[4] Department of Internal Medicine, University Hospital of Larissa, Faculty of Medicine, School of Health Sciences, University of Thessaly, 41110 Larissa, Greece

* Correspondence: petros.cd@gmail.com

PubMed search algorithm 3-11-2024
(("physical activity intervention*"[Title/Abstract] OR "physical activity"[Title/Abstract] OR "aerobic training"[Title/Abstract] OR "fitness"[Title/Abstract] OR "strength exercise"[Title/Abstract] OR "strength training"[Title/Abstract] OR "resistance exercise"[Title/Abstract] OR "resistance training"[Title/Abstract] OR "sport*"[Title/Abstract] OR "exercise"[Title/Abstract] OR "exercise intervention*"[Title/Abstract] OR "motor activity"[Title/Abstract]) AND ("nutrition"[Title/Abstract] OR "paleolithic diet"[Title/Abstract] OR "low carbohydrate diet"[Title/Abstract] OR "carnivore diet"[Title/Abstract] OR "atkins diet"[Title/Abstract] OR "ketogenic diet"[Title/Abstract] OR "keto diet"[Title/Abstract] OR "elimination diet"[Title/Abstract] OR "elemental diet"[Title/Abstract] OR "experimental diet"[Title/Abstract] OR "fasting"[Title/Abstract] OR "mediterranean diet"[Title/Abstract] OR "cretan mediterranean diet"[Title/Abstract] OR "vegetarian diet"[Title/Abstract] OR "vegan diet"[Title/Abstract] OR "lacto vegetarian diet"[Title/Abstract] OR "diet"[Title/Abstract]) AND ("cancer patients"[Title/Abstract] OR "breast cancer"[Title/Abstract] OR "breast carcinoma"[Title/Abstract] OR "breast neoplasm*"[Title/Abstract] OR "breast malignanc*"[Title/Abstract] OR "malignant neoplasm of breast"[Title/Abstract] OR "malignant tumor of breast"[Title/Abstract] OR "mammary cancer"[Title/Abstract] OR "lung cancer"[Title/Abstract] OR "lung malignanc*"[Title/Abstract] OR "lung neoplasm*"[Title/Abstract] OR "bronchogenic malignanc*"[Title/Abstract] OR "bronchogenic cancer"[Title/Abstract] OR "lung adenocarcinoma"[Title/Abstract] OR ("colon"[Title/Abstract] AND "rectum cancer"[Title/Abstract]) OR ("colon"[Title/Abstract] AND "rectum malignanc*"[Title/Abstract]) OR ("colon"[Title/Abstract] AND "rectum neoplasm*"[Title/Abstract]) OR "bowel cancer"[Title/Abstract] OR "bowel neoplasm*"[Title/Abstract] OR "bowel malignanc*"[Title/Abstract] OR "colon cancer"[Title/Abstract] OR "colon neoplasm*"[Title/Abstract] OR "colon malignanc*"[Title/Abstract] OR "rectum cancer"[Title/Abstract] OR "rectum neoplasm*"[Title/Abstract] OR "rectum malignanc*"[Title/Abstract] OR "colorectal cancer"[Title/Abstract] OR "colorectal neoplasm*"[Title/Abstract] OR "colorectal malignanc*"[Title/Abstract] OR "anal cancer"[Title/Abstract] OR "anal neoplasm*"[Title/Abstract] OR

"anal malignanc*"[Title/Abstract] OR "intestinal cancer"[Title/Abstract] OR "intestinal neoplasm*"[Title/Abstract] OR "intestinal malignanc*"[Title/Abstract] OR "prostate cancer"[Title/Abstract] OR "prostate malignanc*"[Title/Abstract] OR "prostate carcinoma"[Title/Abstract] OR "prostate neoplasm*"[Title/Abstract] OR (("male"[MeSH Terms] OR "male"[All Fields]) AND "reproductive system neoplasm*"[Title/Abstract]) OR (("male"[MeSH Terms] OR "male"[All Fields]) AND "reproductive organ cancer"[Title/Abstract]) OR "stomach cancer"[Title/Abstract] OR "stomach neoplasm*"[Title/Abstract] OR "stomach malignanc*"[Title/Abstract] OR "gastric cancer"[Title/Abstract] OR "liver cancer"[Title/Abstract] OR "liver neoplasm*"[Title/Abstract] OR "liver malignanc*"[Title/Abstract] OR "hepatic cancer"[Title/Abstract] OR "hepatic neoplasm*"[Title/Abstract] OR "hepatic malignanc*"[Title/Abstract])) NOT ("animals"[MeSH Terms] NOT "humans"[MeSH Terms])

Table S1: Characteristics of eligible studies. BMI: body mass index; QoL: quality of life; WCRF: world cancer research fund; HIIT: high intensity interval training; NR: not reported; HR: heart rate; RM: repetition maximum; CI: confidence interval; IQR: interquartile range; CDC: centre for disease control; ACSM: American college of sport medicine; HOMA-IR: homeostatic model assessment-insulin resistance.

Code	First author-publication year	Sample characteristics	Cancer type	Physical activity/exercise intervention	Nutrition/diet intervention	Main outcome
1	Brown 2021	Intervention group: n=87; Female=87; Age=60±9; BMI=34.2±6.3; Cancer stage=I-III<br>Control group: n=90; Female=90; Age=59±8.5; BMI=34±5.7; Cancer stage= I-III	Breast	Duration: 52 weeks<br>Type: Resistance + aerobic<br>Content: 9 exercises twice weekly, 2–3 sets, weight permitted 10 repetitions. Moderate-intensity aerobic exercise, 180 min 3–6 days/week	Duration: 52 weeks<br>Type: Hypocaloric diet 10% loss of body weight<br>Content: 7 daily servings of fruits and vegetables, behavioral modification techniques to food shopping and preparation	↓ Body weight and fat mass
2	Brown 2021a	Intervention group: n=87; Female=87; Age=60±9; BMI=34.2±6.3; Cancer stage= I-III<br>Control group: n=90; Female=90; Age=59±8.5; BMI=34±5.7; Cancer stage= I-III	Breast	Duration: 52 weeks<br>Type: Resistance + aerobic<br>Content: 9 exercises twice weekly, 2–3 sets, weight permitted 10 repetitions. Moderate-intensity aerobic exercise, 180 min 4–6 days/week	Duration: 52 weeks<br>Type: Hypocaloric diet 10% loss of body weight<br>Content: 7 daily servings of fruits and vegetables, behavioral modification techniques to food shopping and preparation	↑ QoL
3	Brown 2022	Intervention group: n=67; Female=67; Age=61.5±8.5; BMI=33.7±6.1; Cancer stage= I-III<br>Control group: n=63; Female=63; Age=60.8±7.7; BMI=34.4±5.9; Cancer stage= I-III	Breast	Duration: 52 weeks<br>Type: Resistance + aerobic<br>Content: 9 exercises twice weekly, 2–3 sets, weight permitted 10 repetitions. Moderate-intensity aerobic	Duration: 52 weeks<br>Type: Hypocaloric diet 10% loss of body weight<br>Content: 7 daily servings of fruits and vegetables, behavioral modification techniques to food shopping and preparation	No effect of sex steroid hormones

				exercise, 180 min 3–6 days/week		
4	Brown 2023	Intervention group: n=84; Female=84; Age=59.9±9; BMI=34±6.1; Cancer stage= I-III<br>Control group: n=88; Female=88; Age=58.9±8.4; BMI=34.1±5.8; Cancer stage= I-III	Breast	Duration: 52 weeks<br>Type: Resistance + aerobic<br>Content: 9 exercises twice weekly, 2–3 sets, weight permitted 10 repetitions. Moderate-intensity aerobic exercise, 180 min 3–6 days/week	Duration: 52 weeks<br>Type: Hypocaloric diet 10% loss of body weight<br>Content: 7 daily servings of fruits and vegetables, behavioral modification techniques to food shopping and preparation	↓ Oxidative stress
5	Carayol 2019	Intervention group: n=72; Female=72; Age=51.2±10.9; BMI=25.2±5.4; Cancer stage= I-III<br>Control group: n=71; Female=71; Age=52.1±9.3; BMI=25.8±5.3; Cancer stage= I-III	Breast	Duration: 26 weeks<br>Type: Resistance + aerobic<br>Content:<br>1 resistance session/week, 2-5 sets, 6-12 repetitions. Two moderate intensity 30-45 min aerobic sessions/week, 50–75% maximum heart rate	Duration: 26 weeks<br>Type: Balanced dietary intakes<br>Content: Well-balanced and healthy diet based on WCRF guidelines	Positive changes in a range of psychological, physiological and behavioural outcomes
6	Greenlee 2013	Intervention group:<br>n=22<br>Female=22<br>Age=52.6±8<br>BMI=33.4±6.6<br>Cancer stage= I-III<br>Control group:<br>n=20<br>Female=20<br>Age=48.6±9.6<br>BMI=32.9±5.2<br>Cancer stage= I-III	Breast	Duration: 6 months<br>Type: Resistance + aerobic<br>Content:<br>3 days/week, 30 sec resistance followed by 30 sec aerobic, 25 min followed by 5 min of cool down and stretching	Duration: 6 months<br>Type: Low-fat/calorie-restricted diet<br>Content: 5 small meals, t >2 servings fruit, >3 servings vegetables, 2 l of water, read food labels when choosing foods, and pay attention to intake of total calories, protein, fat, and carbohydrates. reduce caloric intake (1,200 cal/day for 1 to 2 weeks, followed by 1,600 cal/day) and to distribute calorie intake as 45% protein/30% carbohydrates/25% fat.	↓ Body weight

7	Harrigan 2016	Intervention group 1 in person: n=33; Female=33; Age=58.9±7.3; BMI=33.5±6.7; Cancer stage=Survivors<br>Intervention group 2 telephone: n=34; Female=34; Age=60±7.7; BMI=31.8±5.4; Cancer stage= Survivors<br>Control group: n=33; Female=33; Age=58±7.5; BMI=34±7.5; Cancer stage= Survivors	Breast	Duration: 6 months<br>Type: Walking<br>Content: 150/week, moderate-intensity, to increase to 10.000/day	Duration: 6 months<br>Type: Reduced caloric intake<br>Content:<br>fat goal 25% of total energy intake, education on portion sizes, tracking fat grams, reducing simple sugars, and increasing fiber. Reduce energy intake to the range of 1,200 to 2,000 kcal/day based upon baseline weight and to incur an energy deficit of 500 kcal/day.	↓ Body weight and C-reactive protein
8	Jacot 2020	Intervention group: n=180; Female=180; Age=52.66±9.6; BMI=25.72±5.14; Cancer stage= I-IV<br>Control group: n=180; Female=180; Age=52.35±10.09; BMI=25.22±5.30; Cancer stage= I-IV	Breast	Duration: 26 weeks<br>Type: Resistance + aerobic<br>Content:<br>120 min of moderate to vigorous physical activity/week, 50–75% of maximum heart rate, 30-45 min; six main muscle groups 2-5 sets, 6-12 repetitions	Duration: 26 weeks<br>Type: Balanced dietary intake<br>Content: 6 individual nutritional therapeutic education sessions/week for body weight control according to WCRF.	↓ General fatigue score
9	Karimi 2015	Intervention group: n=10; Female=10; Age=47.5±4.6; BMI=31.26±5.1; Cancer stage= I-II<br>Control group: n=10; Female=10; Age=50.4±3.4; BMI=32.77±2.9; Cancer stage= I-II	Breast	Duration: 6 weeks<br>Type: Water-based exercise<br>Content:<br>50-75% of heart rate reserve, in a pool, 4 times/week	Duration: 6 weeks<br>Type: Oral ginger supplement<br>Content: Ginger rhizome powder (750 mg) in 250 ml of water, 4 times/day, with breakfast, lunch, dinner and every afternoon	↓ Inflammation
10	Karimi 2013	Intervention group: n=10; Female=10; Age=48±6; BMI=32±4; Cancer stage= I-II	Breast	Duration: 6 weeks<br>Type: Water-based exercise<br>Content:	Duration: 6 weeks<br>Type: Oral ginger supplement<br>Content: Ginger rhizome powder (750 mg) in 250 ml of water, 4 times/day,	↓ Oxidative stress

		Control group: n=10; Female=10; Age=48±6; BMI=32±4; Cancer stage= I-II		50-75% of heart rate reserve, in a pool, 4 times/week	with breakfast, lunch, dinner and every afternoon	
11	Mefferd 2007	Intervention group: n=47; Female=47; Age=56.3±8.2; BMI=30.7±3.8; Cancer stage= I-IIIA Control group: n=29; Female=29; Age=56.3±8.2; BMI=31.8±4.8; Cancer stage= I-IIIA	Breast	Duration: 16 weeks Type: Resistance + aerobic Content: 1 h/day of moderate to vigorous physical activity. Muscle exercise 2–3 times/week	Duration: 16 weeks Type: Reduction in energy intake Content: A deficit of 500–1000 kcal/day, encouraged to include high-fiber vegetables, whole grains, fruit and protein	↓ Triglycerides, total cholesterol, high density lipoprotein
12	Pakiz 2011	Intervention group: n=44; Female=44; Age=56±9; BMI=30.8±3.8; Cancer stage= Survivors I-IIIA Control group: n=24; Female=24; Age=56±8; BMI=31.3±5.2; Cancer stage= Survivors I-IIIA	Breast	Duration: 16 weeks Type: Physical activity Content: 1 h/day of planned exercise at a moderate level of intensity	Duration: 16 weeks Type: Reduced energy intake Content: An energy deficit of 500-1000 kcal/day by individualized diet modification that emphasized reduced energy density of the overall diet	Favorable changes in cytokine levels in association with weight loss
13	Puklin 2021	Intervention group: n=91; Female=91; Age=59±7.3; BMI=NR; Cancer stage= I-IIIA Control group: n=58; Female=58; Age=56.3±8.4; BMI=NR; Cancer stage= I-IIIA	Breast	Duration: 6 months Type: Physical activity Content: 150/week of moderate-intensity activity, daily step count to 10000 steps/day	Duration: 6 months Type: Reduced energy intake Content: A reduction of 1200-2000 kcal/day based upon baseline weight and to incur an energy deficiency of 500 kcal/day. Education on portion size, tracking fat grams, reducing simple sugars, increasing fiber, and incorporating mindful eating techniques	Weight loss, achieved through a lifestyle intervention, is associated with higher ghrelin levels in breast cancer survivors
14	Puklin 2023	Intervention group: n=102; Female=102; Age=57±10.7;	Breast	Duration: 6 months	Duration: 6 months Type: Book and videos guidance	

		BMI=32.3±4.8; Cancer stage= 0-IIIC<br>Control group: n=103; Female=103; Age=57.9±10; BMI=32.2±5.3; Cancer stage=0-IIIC		Type: Home-based physical activity programme<br>Content: 150 min/week of brisk walking (or other moderate intensity activity of choice), aiming at 10.000 steps/day	Content: Guidance on increasing fruit and vegetable servings, reducing energy intake (1200 to 2000 kcal/day), increasing fiber, and limiting dietary fat (<25% of total energy)	↓ Body weight
15	Reeves 2016	Intervention group: n=45; Female=45; Age=56.4±9; BMI=30.6±4.3; Cancer stage=I-III<br>Control group: n=45; Female=45; Age=54.3±8.4; BMI=31.4±4.2; Cancer stage= I-III	Breast	Duration: 6 months<br>Type: Self-guided- increase of general activity<br>Content: 30 min/day (210 min/week of general activity, for example brisk walking)	Duration: 6 months<br>Type: Weight loss diet<br>Content: Guidance to achieve a kJ goal between 5000-7500 kJ/day (aiming for 2000 kJ energy deficit), Participants were encouraged to aim for total fat intake <30% of energy, saturated fat <7% of energy, five servings/day of vegetables and two servings/day of fruit.	↓ Body weight and fat mass
16	Reeves 2021	Intervention group: n=79; Female=79; Age=55.9±9.1; BMI=31.4±4.9; Cancer stage= Survivors<br>Control group: n=80; Female=80; Age=54.9±9.3; BMI=31.5±5.2; Cancer stage= Survivors	Breast	Duration: 12 months<br>Type: Resistance + aerobic<br>Content: moderate-to-vigorous intensity aerobic, 210 min/week and 2–3 resistance exercise sessions/week were encouraged	Duration: 12 months<br>Type: telephone guidance for weight loss of 5–10%<br>Content: Energy intake reduction (1200–1500 kcal/day), reduction of saturated fat (<7% total energy, increase of eating vegetables and fruit (5 and 2 servings/day, respectively), limit of alcohol (≤1 serving/day)	↓ Body weight, fat mass, metabolic syndrome risk score, plasma glucose, waist circumference, musculoskeletal pain.<br>Improved physical QoL and body image.
17	Sanft 2023	Intervention group: n=87; Female=87; Age=52.3±11.3; BMI=29.5±7; Cancer stage= I-III<br>Control group: n=86; Female=86; Age=53.3±10.9; BMI=29.8±6.6; Cancer stage= I-III	Breast	Duration: 3.3±1.2 months<br>Type: Resistance + aerobic<br>Content: 4 weekly sessions in the first month, two biweekly sessions for months 2	Duration: 3.3±1.2 months<br>Type: Plant-based diet<br>Content: A combination of ≥5 fruits and/or vegetable servings/day, ≥25 g/day of fiber, <30 g/day of added, sugars, ≤18 ounces/week of red meat, limited consumption of processed	Positive association oft he intervention with pathologic complete response

				and 3, and monthly sessions thereafter. ≥150 min/week of moderate- to vigorous-intensity physical activity or 75 min/week of vigorous-intensity physical activity and twice-weekly resistance training	foods, and alcohol consumption ≤1 drink/day	
18	Sanft 2018	Intervention group: n=93; Female=93; Age=58.7±7.2; BMI=31.9±5.7; Cancer stage= I-III<br>Control group: n=58; Female=58; Age=56.3±8.4; BMI=34.6±6.7; Cancer stage= I-III	Breast	Duration: 6 months<br>Type: Home based exercise (walking)<br>Content: 150 min/week of moderate-intensity activity and 10.000 steps/day	Duration: 6 months<br>Type: Reduced caloric intake<br>Content: Reducing calories to 1200–2000 kcal/day, adjusted for baseline weight, and reducing dietary fat to <25% of total energy intake	↑ Telomere length
19	D'Alonzo 2021	Intervention group: n=55; Female=55; Age=60.2±9.2; BMI=33.4±5.8; Cancer stage= Survivors<br>Control group: n=51; Female=51; Age=60.5±8.9; BMI=33.3±5.3; Cancer stage= Survivors	Breast	Duration: 12 months<br>Type: Home-based resistance + aerobic<br>Content: 2 weight training sessions and 180 min of aerobic exercise/week. First six weeks, participants received intervention in person, on-site weekly. From weeks 7-52, participants received monthly in-person sessions at the study site, in addition to performing 2 weight training and 6 aerobic	Duration: 12 months<br>Type: Caloric restriction via group meetings<br>Content: During the first 20 weeks, daily caloric intake was restricted to 1200–1500 kcal/day. From weeks 20–24 participants were encouraged to transition purchasing their own food from the grocery store while maintaining 1200–1500 kcal/day. During the following 28 weeks, participants increased their caloric intake to 1700–2000 kcal/day with the goal of maintaining the weight they had lost in the initial 24 weeks	↓ insulin, C-peptide, HOMA-IR, and HOMA2 beta-cell function

				exercise sessions/week at home.		
20	Schmitz 2019	Intervention group: n=87; Female=87; Age=60±9; BMI=34.2±6.3; Cancer stage= Survivors<br>Control group: n=90; Female=90; Age=59±8.5; BMI=34±5.7; Cancer stage= Survivors	Breast	Duration: 52 weeks<br>Type: Walking + Resistance exercises<br>Content: Walking goals/week were: 90 min for weeks 1-3, 120 min for week 4, 150 min for weeks 5-6, and 180 min thereafter. Resistance progression was symptom-limited by 0.45-0.90 kg every 2 weeks, with an upper limit of 9.45 kg, twice/session during weeks 1-6 and 3 times/session thereafter.	Duration: 52 weeks<br>Type:<br>Content: Weeks 1-20: Participants followed a meal replacement program that included 7 servings of fruits and vegetables daily. During weeks 21-24 lessons shopping for and preparing their own food. From weeks 25-52, groups met monthly for weigh-ins and additional behavioural modification lessons that focused on weight maintenance. The weight loss goal was 10% of baseline body weight.	The intervention did not affect breast cancer–related lymphedema outcomes
21	Scott 2013	Intervention group: n=47; Female=47; Age=55.6±10.2; BMI=29.6±3.5; Cancer stage= I-III<br>Control group: n=43; Female=43; Age=55.9±8.9; BMI=31.1±5.6; Cancer stage= I-III	Breast	Duration: 6 months<br>Type: Resistance + aerobic<br>Content: 3 times/week supervised, 30 min of aerobic exercise (65–85 % age-predicted maximum heart rate) using treadmill, cross-trainer, cycle ergometer, and/or rowing ergometer, followed by 10–15 min of muscle-strengthening exercises using	Duration: 6 months<br>Type: Hypocaloric, healthy eating program<br>Content: One-to-one individualized dietary advice and written information on portion sizes from common foods in each food group and a healthy eating plan. Reduce total daily calorie intake to 600 kcal below the calculated energy requirements, estimated steady weight loss of up to 0.5 kg/week. Weekly small-group nutrition education seminars included topics such as dietary fat intake, hydration, achieving a healthy	The intervention had positively impact upon health outcomes influencing long-term prognosis in recovering from early-stage breast cancer

				resistance bands, hand weights, and stability balls.	balanced diet, and alcohol consumption.	
22	Baguley 2022	Intervention group: n=12; Male=12; Age=66.6±7.6; BMI=27.4±3; Cancer stage=NR<br>Control group: n=11; Male=11; Age=65.1±7.9; BMI=30.6±2.9; Cancer stage=NR	Prostate	Duration: Weeks 12-20<br>Type: HIIT<br>Content: Air- and magnetically-braked cycle ergometer, 3 times/week, 10 min warm up at 50–70% HRpeak, 4 x 4 min cycling at 85–95% HRpeak. Each 4 min interval with a 3 min period of active recovery at 50–70% HRpeak, totalling 38 min for the session.	Duration: 20 weeks<br>Type: Mediterranean diet<br>Content: Total energy intake decrement using the Harris-Benedict predicted energy requirements, with a dietary composition of 45-65% carbohydrate, 20-35% fat, saturated fat <10% total energy intake, and 15-25% protein sources. A dietary energy reduction of 2000-4000 kJ/day was consulted at baseline if body composition and diet intake were classified average or poor	The intervention increased cardiorespiratory fitness and reduced body weight
23	Bourke 2014	Intervention group: n=50; Male=50; Age=71±6; BMI=29.3±4.4; Cancer stage=NR<br>Control group: n=50; Male=50; Age=71±8; BMI=28.1±4.1; Cancer stage=NR	Prostate	Duration: 12 weeks<br>Type: Resistance + aerobic<br>Content: Twice a week from weeks 1–6, and once per week from weeks 7–12. Aerobic exercise 30 min at 55–75% of age predicted maximum heart rate or 11–13 on the Borg Rating of Perceived Exertion scale, using stationary cycles, rowing ergometers, and treadmills. 2-4 sets and 8–12 repetitions of resistance exercises	Duration: 12 weeks<br>Type: Dietary advice<br>Content: A nutrition advice pack was provided, and small-group healthy eating seminars, lasting approximately 20 min, were carried out every 2 weeks	The intervention improved QoL that was not maintained postintervention. No effect on blood pressure occurred.

				beginning at 60% of one repetition max with progression through increasing volume before weight was increased. This was undertaken twice a week from weeks 1–6, and once per week from weeks 7–12		
24	Bourke 2011	Intervention group: n=25; Male=25; Age=71.3±6.4; BMI=28±3.2; Cancer stage=NR<br>Control group: n=25; Male=25; Age=72.2±7.7; BMI=27.4±2.7; Cancer stage=NR	Prostate	Duration: 12 weeks<br>Type: Resistance + aerobic<br>Content: 30 min aerobic exercise of 55%–85% of age-predicted maximum heart rate and/or ratings of perceived exertion, 11-15/fairly light to hard, on the Borg Rating of Perceived Exertion. 2-4 sets body weight resistance and free weights targeting large skeletal muscle groups, twice weekly for the initial 6 weeks and then once weekly for the following 6 weeks. Also, self-directed exercise (e.g., brisk walking, cycling, and gym exercise) for at least one 30-min/session/week	Duration: 12 weeks<br>Type: Dietary advice<br>Content: Reduction of saturated fat and refined carbohydrate and increase of dietary fiber intake with moderation of alcohol, in seminars of 15-20 min.	The intervention had positive impact on health behaviours

				during the initial 6 weeks and at least 2 sessions/week for the final 6 weeks		
25	Chaplow 2020	Intervention group: n=16; Male=16; Age=67.9±7.9; BMI=29±4.4; Cancer stage=NR<br>Control group: n=16; Male=16; Age=64.3±6.1; BMI=31.4±5.9; Cancer stage=NR	Prostate	Duration: 3 months<br>Type: Resistance + aerobic<br>Content: combination of resistance and aerobic exercise, 1 hour supervised twice/week	Duration: 3 months<br>Type: Counselling according to 2010–2015 Dietary Guidelines for Americans, the American Heart Association/American College of Cardiology, and the World Cancer Research Fund/American Institute for Cancer Research<br>Content: 8 group-based nutritional counselling, individualized phone sessions. Advice about a diet rich in whole grains, vegetables, and fruits, limited consumption of processed high fat, low nutrient dense foods, reduced intake of red and processed meats and overall caloric intake levels that promote achieving and maintaining a healthy body weight and avoiding weight gain	The intervention led to superior preservation of lean tissue and improvement in adiposity relative to standard-of-care treatment
26	Daubenmier 2006	Intervention group: n=44; Male=44; Age=64.8±7.1; BMI=26±4.2; Cancer stage=Early<br>Control group: n=49; Male=49; Age=66.5±7.6; BMI=25.9±4.2; Cancer stage= Early	Prostate	Duration: 12 months<br>Type: Not defined<br>Content: 3 hours/week moderate exercise and 1 hour/day of stress management practice (ie, mindful stretching, breathing techniques, meditation, visualization, progressive relaxation)	Duration: 12 months<br>Type: Vegan diet<br>Content: A plant-based vegan diet with 10% of total calories from fat	↑ QoL
27	Focht 2018	Intervention group: n=16; Male=16; Age=69.4±9;	Prostate	Duration: 12 weeks	Duration: 12 weeks	

		BMI=28.5±9.05; Cancer stage=NR<br>Control group: n=16; Male=16; Age=64.5±8.6; BMI=31.5±6.23; Cancer stage=NR		Type: Resistance + aerobic<br>Content: 3 sets, 8–12 repetition maximum (8RM–12RM) and a rating of perceived exertion 1–10 ranging from 3 (Moderately Hard) to 6 (Hard), 9 different exercises (leg extension, leg curl, chest press, lateral pull-down, overhead press, triceps extension, bicep curl, calf raises, and abdominal crunch), 1–2 min rest interval. Aerobic exercise stimulus of 10–20 min of exercise performed at a rating of perceived exertion (1–10) ranging from 3 (fairly light) to 4 (moderately hard) on the participant's choice of a treadmill, stationary cycle, or elliptical trainer. Home-based exercise and decrease sedentary time towards 150 min of physical activity/week and 10,000 steps/day.	Type: Nutrition advise consistent with the dietary objectives recommended by 2010–2015 Dietary Guidelines for Americans, the American Heart Association/American College of Cardiology, the American Institute of Cancer Research, and the American Cancer Society.<br>Content: 6 small group sessions (20–30 min) conducted once/week immediately following a centre-based exercise session during months 1–2. Participants also received four brief 20-min individualized activity counselling sessions conducted via phone calls in months 1–3.	↑ clinically meaningful improvements in mobility performance, muscular strength, and body composition

28	Frattaroli 2008	Intervention group: n=44; Male=44; Age=66±8 (both groups); BMI=NR; Cancer stage=Early<br>Control group: n=49; Male=49; Age=66±8 (both groups); BMI=NR; Cancer stage= Early	Prostate	Duration: 2 years<br>Type: Moderate aerobic exercise<br>Content: Walking 30 min/day, 6 days/week	Duration: 2 years<br>Type: Vegan diet<br>Content: Predominantly fruits, vegetables, whole grains (complex carbohydrates), legumes, and soy products, was low in simple carbohydrates, and included approximately 10% of calories from fat	The intervention may lead to avoid or delay conventional treatment
29	Freedland 2019	Intervention group: n=11; Male=11; Age=66 (CI:61-76); BMI=28.9 (CI:27.5-38.7); Cancer stage= NR<br>Control group: n=18; Male=18; Age=66 (CI:56-70); BMI=29.1 (CI:27-32); Cancer stage=NR	Prostate	Duration: 6 months<br>Type: Walking<br>Content: Walking ≥30 min/day for ≥5 days/week.	Duration: 6 months<br>Type: Low-carbohydrate diet counselling via phone<br>Content: Limit of carbohydrate intake to ≤20g/day. Participants were provided a list of low-carbohydrate foods (e.g., greens, lean meat, and seafood) and a list of moderate/high carbohydrate foods to limit (e.g., bread, pasta, legumes, and starchy vegetables). Grains and starchy vegetables are high in carbohydrates (e.g., 1 slice of bread contains about 12g carbohydrate); thus, these food items are on the limit list. Sample menus and recipes were also provided.	↓ Insulin resistance
30	Fruge 2018	Intervention group: n=11; Male=11; Age=60.9 (both groups); BMI=30.2 (IQR:28.2-33.5); Cancer stage= NR<br>Control group: n=11; Male=11; Age=60.9 (both groups); BMI=31.3 (IQR:27.1-33.5); Cancer stage= NR	Prostate	Duration: 13 weeks<br>Type: Advise to exercise<br>Content: Semi-weekly in-person and telephone contact with an exercise physiologist, 30 min/day to promote weight loss of 0.91 kg/week	Duration: 13 weeks<br>Type: Nutrition advise<br>Content: Semi-weekly in-person and telephone contact with a registered dietitian nutritionist, calorie-restricted diet to promote weight loss of 0.91 kg/week	No relationship of the intervention with microbes

31	Hebert 2012	Intervention group: n=47; Male=47; Age=69.7±8.8; BMI=28.9±4.9; Cancer stage=NR<br>Control group: n=54; Male=54; Age=71.1±8.1; BMI=29.4±5.6; Cancer stage=NR	Prostate	Duration: 6 months<br>Type: Not specified<br>Content: CDC/ACSM recommendations of days/week, a goal of leisure-time activity currently recommended for attaining health benefits. 45 min training sessions, included stretching (5 min), warm-up (3–5 min), and 30 min of the actual exercise (e.g., brisk walking), followed by a cool-down period of ''active'' recovery	Duration: 6 months<br>Type: Not specified<br>Content: Food-related goals including decreasing meat and dairy consumption while increasing consumption of whole grains, soybeans and soybean products, other beans, and vegetables	The intervention had positive health changes in a number of lifestyle parameters
32	O'Neill 2015	Intervention group: n=47; Male=47; Age=69.9±6.8; BMI=29.9±4.5; Cancer stage=I-IV<br>Control group: n=47; Male=47; Age=69.9±7; BMI=29.7±4.6; Cancer stage= I-IV	Prostate	Duration: 6 months<br>Type: Physical activity<br>Content: Walking at a brisk pace for at least 30 min/day, five or more days/week in line with UK physical activity guidelines	Duration: 6 months<br>Type: Diet commensurate with UK healthy eating guidelines<br>Content: ≥5 servings of vegetables and fruits/day, 30–35% of total energy from fat and <10% energy from saturated fat/day, 10% of energy from polyunsaturated fat/day, limited consumption of processed meats, 25–35 g of fibre/day, ≤28 units/week of alcohol and limited intake of foods high in salt and/or sugar	The intervention minimised the adverse body composition changes associated with androgen deprivation therapy
33	Wilson 2021	Intervention and control group (cross-over design): n=14; Male=14; Age=72±9 BMI=34.4±6.4; Cancer stage=NR	Prostate	Duration: 12 weeks<br>Type: Resistance + aerobic<br>Content: 60 min moderate-to-vigorous	Duration: 12 weeks<br>Type: Energy deficit and protein supplementation<br>Content: Energy deficit of 500–1000 kcal, reduction of alcohol and foods	The intervention reduced fat mass, maintain lean mass, and improved muscle strength and cardiorespiratory fitness

				intensity aerobic exercise/day, defined as an RPE of 3–8 on the Borg 1–10 scale; resistance training 6-12 repetition maximum over 1–4 sets/exercise	containing refined sugar; a 40g whey protein supplement 3 times/week after resistance exercise	
34	Ho 2020	Intervention group: n=55; Female=18; Age=63.2±11.4; BMI=23.8±3.3; Cancer stage=survivors<br>Control group: n=56; Female=26; Age=64.9±9.4; BMI=23.9±3.6; Cancer stage= survivors	Colorectal	Duration: 12 months<br>Type: Moderate-vigorous physical activity<br>Content: Target of 60 min of moderate-vigorous physical activity 5 days/week	Duration: 12 months<br>Type: Consultation every 2 weeks<br>Content:<br>Interviews and phone calls, reduction of red/processed meat consumption <5 serving/week, to limit refined grains to 2 servings/day	↑ QoL and Depression
35	Lee 2018	Intervention group: n=55; Female=18; Age=63.2±11.4; BMI=23.8±3.3; Cancer stage= survivors<br>Control group: n=56; Female=26; Age=64.9±9.4; BMI=23.9±3.6; Cancer stage=survivors	Colorectal	Duration: 12 months<br>Type: Moderate to vigorous<br>Content: Progressively increase their physical activity levels to 60 minutes of moderate-vigorous 5 days/week	Duration: 12 months<br>Type: Consultation face-to-face interviews and phone calls<br>Content: High dietary fibre, low red and processed meat and refined grain	The intervention modified dietary and physical activity habits
36	Demark-Wahnefried 2012	Intervention group: n=243; Female=132; Age=73±5.2; BMI=29.1(mean)±0.2(SE); Cancer stage=survivors<br>Control group: n=245; Female=138; Age=72.9±5; BMI=29.1(mean)±0.2(SE); Cancer stage=survivors	Breast, prostate, or colorectal	Duration: 12 months<br>Type: Resistance + aerobic<br>Content: 15 minutes of strength-training exercise every other day, 30 min of endurance exercise/day	Duration: 12 months<br>Type: Healthy caloric-restricted diet<br>Content: Daily consumption of 7 servings (women) or 9 servings (men) of fruits and vegetables, restriction of saturated fat <10% of energy intake, and modest weight loss of less than 0.5 kg/week	Favour effect of the intervention on lifestyle behaviour
37	Morey 2009	Intervention group: n=319; Female=172; Age=73±5;	Breast, prostate,	Duration: 12 months<br>Type: Resistance + aerobic	Duration: 12 months<br>Type: Healthy caloric-restricted diet	The intervention improved the self-reported functional decline

		BMI=29.1±3.3; Cancer stage=survivors<br>Control group: n=322; Female=177; Age=73.1±5.1; BMI=29.2±3.6; Cancer stage=survivors	or colorectal	Content: 15 minutes of strength-training exercise every other day, 30 minutes of endurance exercise/day	Content: Consumption of at least seven servings (for women) or nine servings (for men) of fruits and vegetables per day;11 restriction of saturated fat to less than 10% of energy intake and a 10% weight loss	
38	Ferreira 2021	Intervention group: n=24; Female=11; Age=67(median)(63.3-72 IQR); BMI=26.6(median)(23-32.6 IQR); Cancer stage= I-IIIa<br>Control group: n=10; Female=5; Age=69(median)(66.8-73.3 IQR); BMI=28.3(median)(23.6-31.2 IQR); Cancer stage= I-IIIa	Lung	Duration: 4 weeks<br>Type: Resistance + aerobic<br>Content: 10 resistance exercises targeting major muscle groups, 1–2 sets of 8–15 repetitions. Aerobic moderate intensity 90% of the workload, 30 min.	Duration: 4 weeks<br>Type: Protein diet<br>Content: Protein intake of >1.2 g/kg/day and energy of 25–30 kcal/kg/day + leucine doses pre-mixed in powder form, in unlabelled containers to dilute in 125 mL water; a daily dose of fruit-flavoured fish oil containing vitamin D, a dosing cup, pre-marked to 10 mL, providing 1500 mg EPA, 1000 mg DHA and 2000 IU vitamin D3	The intervention deemed feasible for the patients.

Figure S1: PRISMA flow diagram.

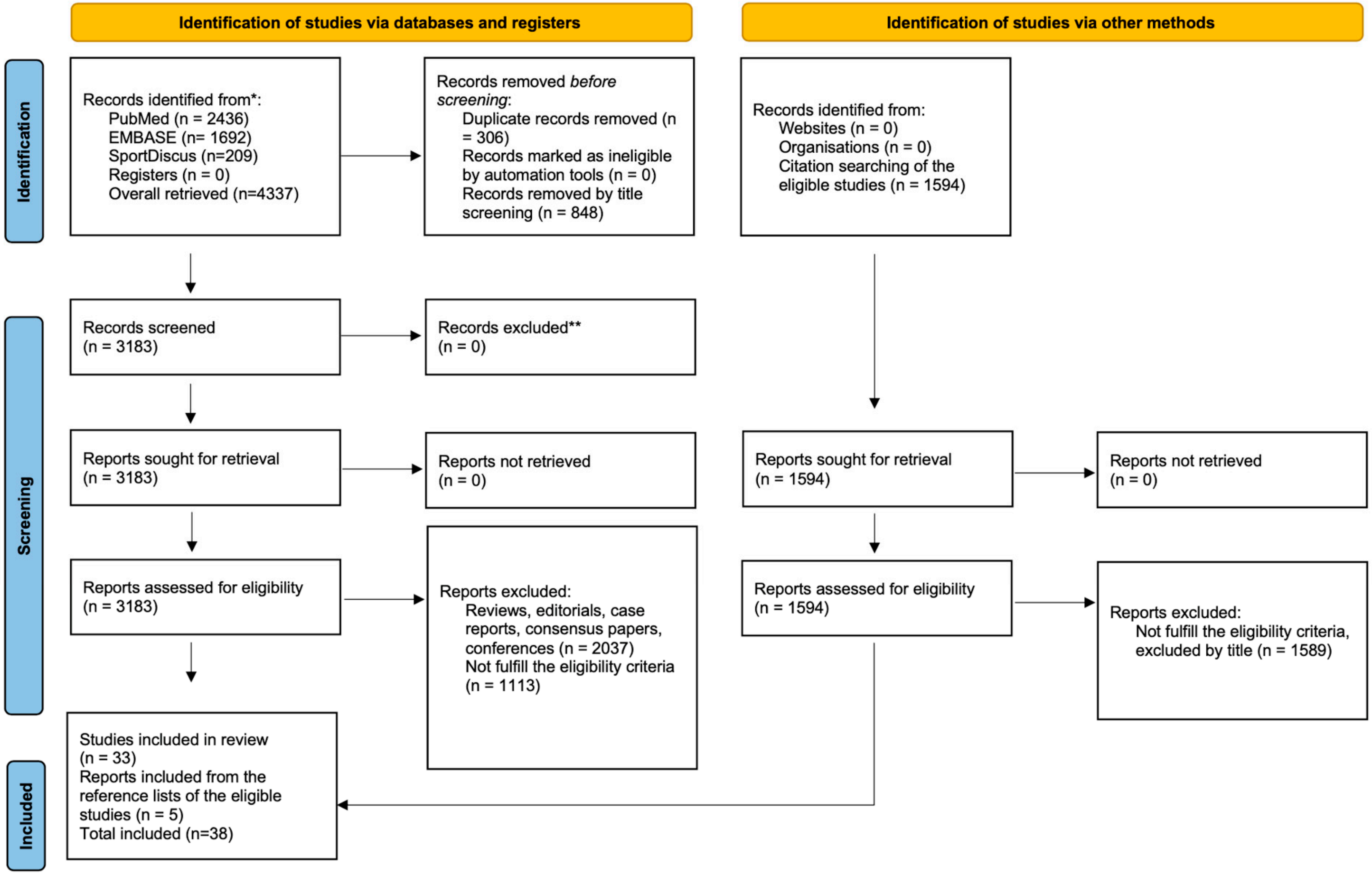

Table S2: Risk of bias assessment results

Study ID	Author	Randomization process	Deviations from intended interventions	Missing outcome data	Measurement of the outcome	Selection of the reported result	Overall Bias
1	Baguley 2022	+	+	+	-	+	-
2	Brown 2021	-	+	+	-	-	-
3	Brown 2021a	?	-	+	-	?	-
4	Brown 2022	-	?	+	+	-	-
5	Brown 2023	-	-	+	+	?	-
6	Bourke 2014	+	+	+	+	+	+
7	Bourke 2011	?	+	+	-	?	-
8	Carayol 2019	?	?	+	-	-	-
9	Chaplow 2020	?	+	+	+	-	-
10	Daubenmier 2006	+	+	+	+	?	?
11	Demark-Wahnefried 2012	?	+	+	-	-	-
12	D'Alonzo 2021	-	+	+	+	?	-
13	Ferreira 2021	-	+	+	-	-	-
14	Focht 2018	+	+	+	+	?	?
15	Frattaroli 2008	?	-	+	-	+	-
16	Freedland 2019	+	?	+	-	?	-
17	Frugé 2018	+	+	+	?	+	?
18	Greenlee 2013	+	?	+	+	?	?
19	Harrigan 2016	?	?	?	-	-	-
20	Hébert 2012	+	+	+	+	+	+
21	Ho 2020	-	+	+	+	+	-
22	Jacot 2020	-	+	?	+	-	-
23	Karimi 2015	?	?	+	+	?	?
24	Karimi 2013	?	?	+	+	?	?
25	Lee 2018	?	+	+	-	+	-
26	Mefferd 2007	-	-	+	-	+	-
27	Morey 2009	-	+	-	-	-	-
28	O'Neill 2015	+	?	+	+	-	-
29	Pakiz 2011	+	?	+	-	+	-
30	Puklin 2021	+	+	+	-	+	-
31	Puklin 2023	-	?	-	-	-	-
32	Reeves 2016	+	+	+	-	-	-
33	Reeves 2021	?	?	+	+	?	-
34	Sanft 2023	+	-	+	-	?	-
35	Sanft 2018	?	?	+	-	+	-
36	Schmitz 2019	+	?	+	-	?	-
37	Scott 2013	+	?	+	+	-	-
38	Wilson 2021	-	+	+	-	-	-

Figure S2: Funnel plot on the effects of diet/nutrition and physical activity/exercise intervention on body mass index.

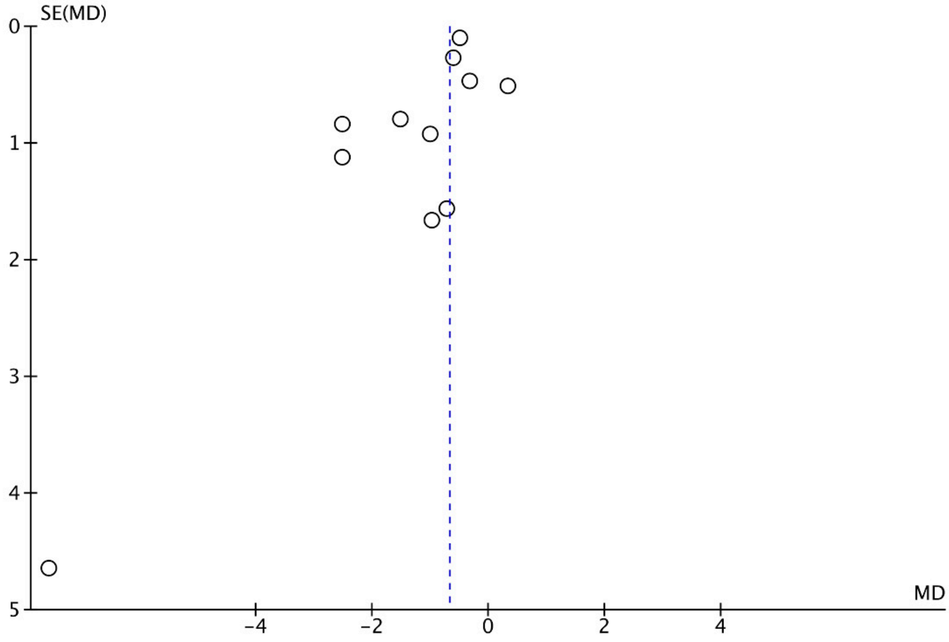

Figure S3: Subgroup analysis of the effects of diet/nutrition and physical activity/exercise intervention on body mass index.

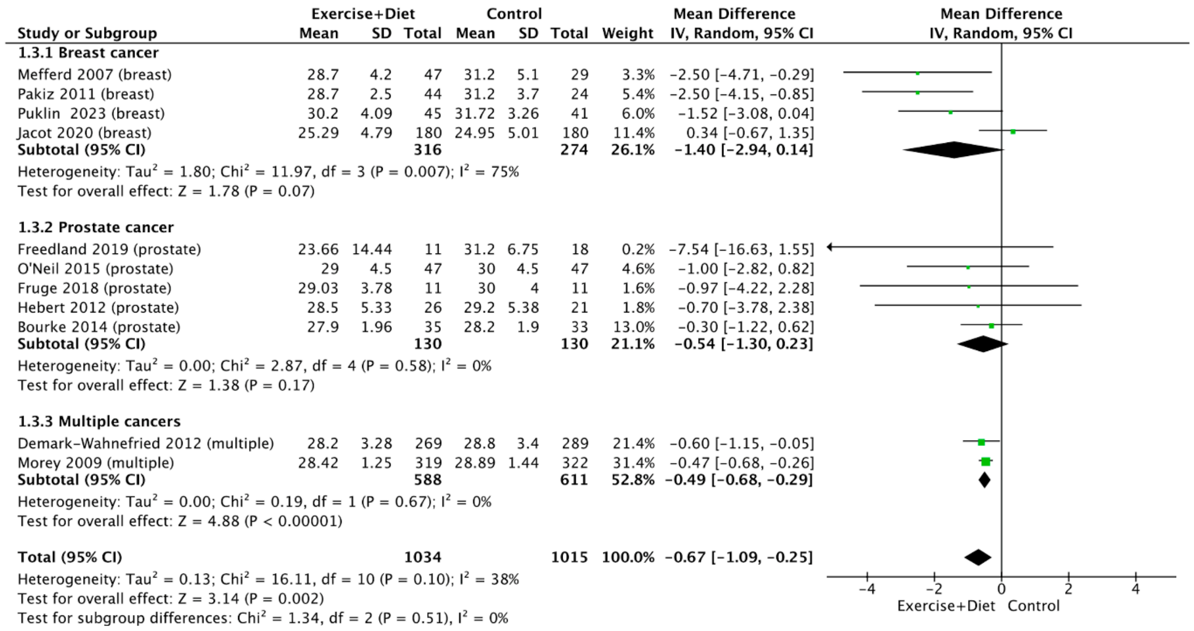

Figure S4: Funnel plot on the effects of diet/nutrition and physical activity/exercise intervention on body weight.

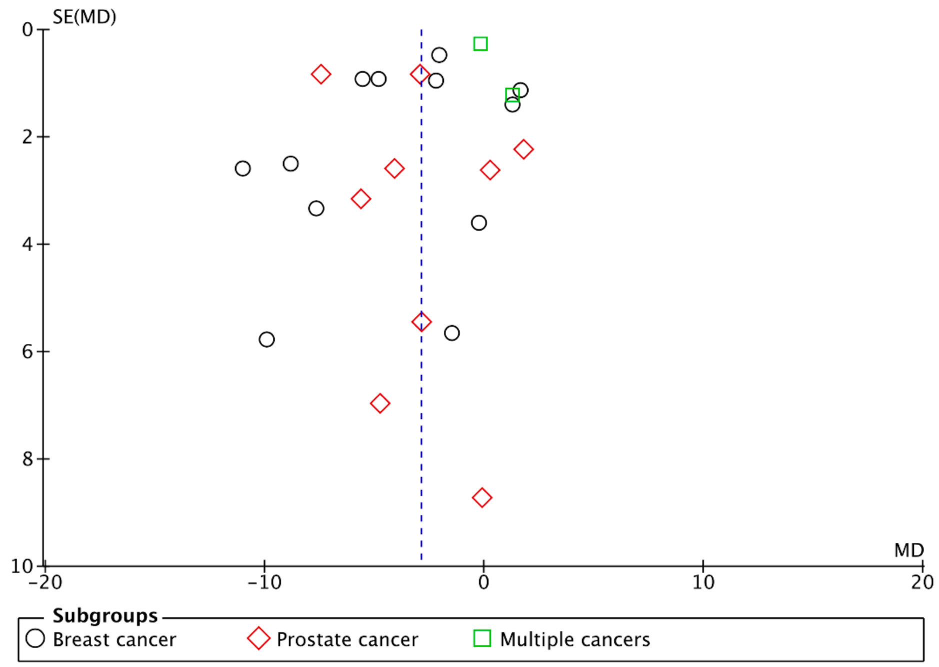

Figure S5: Funnel plot on the effects of diet/nutrition and physical activity/exercise intervention on fat mass.

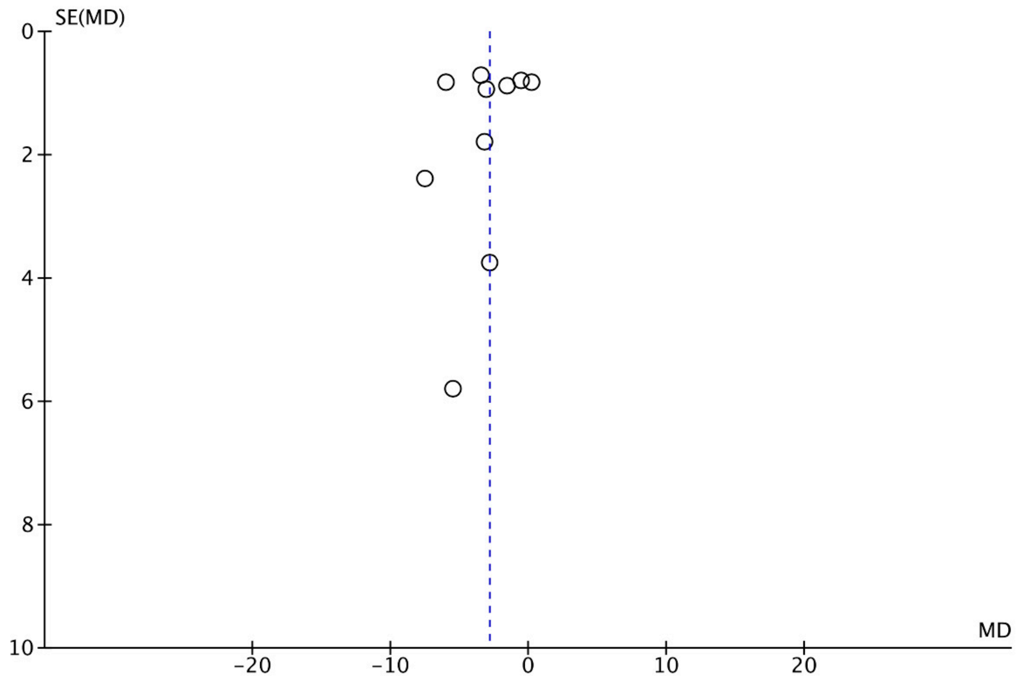

Figure S6: Subgroup analysis of the effects of diet/nutrition and physical activity/exercise intervention on fat mass.

Study or Subgroup	Exercise+Diet Mean	SD	Total	Control Mean	SD	Total	Weight	Mean Difference IV, Random, 95% CI
**1.7.1 Breast cancer**								
Mefferd 2007 (breast)	31.2	9	47	38.7	10.8	29	6.6%	−7.50 [−12.20, −2.80]
Brown 2021 (breast)	36.48	4.94	87	39.96	4.84	90	13.6%	−3.48 [−4.92, −2.04]
Reeves 2016 (breast)	28.9	4.28	45	31.9	4.62	45	12.8%	−3.00 [−4.84, −1.16]
Reeves 2021 (breast)	36.69	4.37	63	37.21	4.27	54	13.4%	−0.52 [−2.09, 1.05]
Greenlee 2013 (breast)	34.3	2.4	22	34	2.7	17	13.2%	0.30 [−1.33, 1.93]
**Subtotal (95% CI)**			**264**			**235**	**59.6%**	**−2.28 [−4.23, −0.33]**
Heterogeneity: $Tau^2 = 3.74$; $Chi^2 = 21.42$, df = 4 (P = 0.0003); $I^2 = 81\%$								
Test for overall effect: Z = 2.29 (P = 0.02)								
**1.7.2 Prostate cancer**								
Chaplow 2020 (prostate)	32.61	2.75	16	38.58	1.7	14	13.3%	−5.97 [−7.59, −4.35]
Focht 2018 (prostate)	34.92	9.95	14	40.38	17.03	11	1.8%	−5.46 [−16.79, 5.87]
O'Neil 2015 (prostate)	26.9	7.8	47	30.1	9.6	47	8.8%	−3.20 [−6.74, 0.34]
Wilson 2021 (prostate)	37	9.5	14	39.8	10.3	14	3.6%	−2.80 [−10.14, 4.54]
Baguley 2022 (prostate)	27.8	2.03	11	29.3	2.02	10	13.0%	−1.50 [−3.23, 0.23]
**Subtotal (95% CI)**			**102**			**96**	**40.4%**	**−3.62 [−6.30, −0.94]**
Heterogeneity: $Tau^2 = 5.18$; $Chi^2 = 13.96$, df = 4 (P = 0.007); $I^2 = 71\%$								
Test for overall effect: Z = 2.65 (P = 0.008)								
**Total (95% CI)**			**366**			**331**	**100.0%**	**−2.85 [−4.45, −1.25]**
Heterogeneity: $Tau^2 = 4.33$; $Chi^2 = 43.30$, df = 9 (P < 0.00001); $I^2 = 79\%$								
Test for overall effect: Z = 3.49 (P = 0.0005)								
Test for subgroup differences: $Chi^2 = 0.63$, df = 1 (P = 0.43), $I^2 = 0\%$								

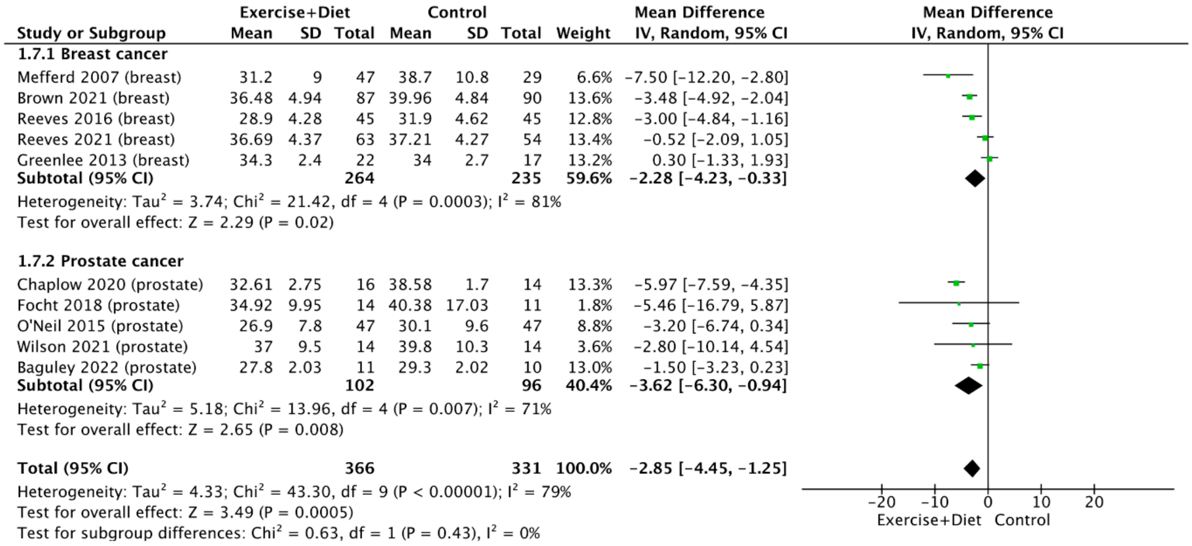

Figure S7: Funnel plot of the effects of diet/nutrition and physical activity/exercise intervention on fat free mass.

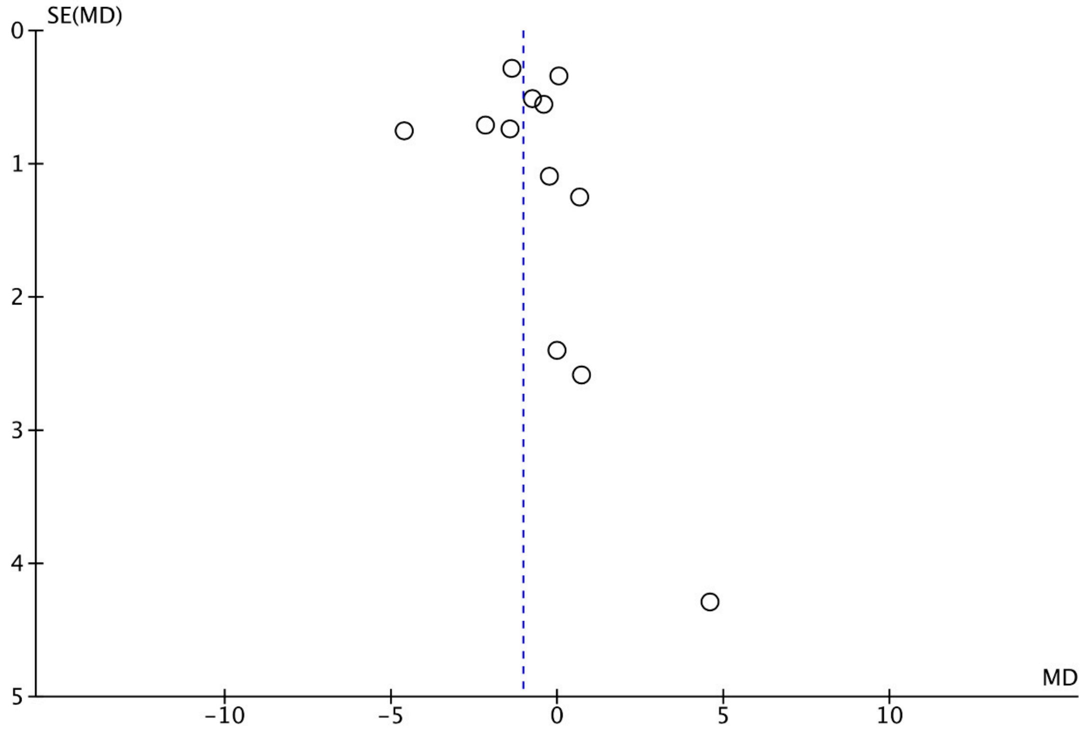

Figure S8: Subgroup analysis of the effects of diet/nutrition and physical activity/exercise intervention on fat free mass.

Study or Subgroup	Exercise+Diet Mean	SD	Total	Control Mean	SD	Total	Weight	Mean Difference IV, Random, 95% CI
**1.9.1 Breast cancer**								
Harrigan 2016 (breast-in telephone)	43.73	2.79	24	48.34	2.84	31	9.8%	−4.61 [−6.11, −3.11]
Harrigan 2016 (breast)	46.21	2.79	30	48.34	2.84	31	10.2%	−2.13 [−3.54, −0.72]
Reeves 2021 (breast)	41.73	1.59	64	43.08	1.56	54	13.9%	−1.35 [−1.92, −0.78]
Greenlee 2013 (breast)	46.9	1.5	21	47.3	1.9	17	11.6%	−0.40 [−1.51, 0.71]
Mefferd 2007 (breast)	40.8	4.6	47	41	4.7	29	7.2%	−0.20 [−2.36, 1.96]
Brown 2021 (breast)	49.1	2.24	87	49.04	2.37	90	13.5%	0.06 [−0.62, 0.74]
**Subtotal (95% CI)**			**273**			**252**	**66.2%**	**−1.40 [−2.54, −0.25]**
Heterogeneity: Tau² = 1.64; Chi² = 37.17, df = 5 (P < 0.00001); I² = 87%								
Test for overall effect: Z = 2.39 (P = 0.02)								
**1.9.2 Prostate cancer**								
Baguley 2022 (prostate)	52	1.69	11	53.4	1.69	10	10.1%	−1.40 [−2.85, 0.05]
Chaplow 2020 (prostate)	56.51	1.5	14	57.23	1.29	16	12.1%	−0.72 [−1.73, 0.29]
Wilson 2021 (prostate)	55.9	6.2	14	55.9	6.5	14	2.4%	0.00 [−4.71, 4.71]
O'Neil 2015 (prostate)	59.8	6.6	45	59.1	5.2	45	6.2%	0.70 [−1.75, 3.15]
Focht 2018 (prostate)	57.62	7.38	14	56.87	5.57	11	2.2%	0.75 [−4.33, 5.83]
**Subtotal (95% CI)**			**98**			**96**	**33.0%**	**−0.72 [−1.48, 0.04]**
Heterogeneity: Tau² = 0.00; Chi² = 2.55, df = 4 (P = 0.64); I² = 0%								
Test for overall effect: Z = 1.84 (P = 0.07)								
**1.9.3 Lung cancer**								
Ferreira 2021 (lung)	50.5	11.7	14	45.9	7.2	6	0.9%	4.60 [−3.81, 13.01]
**Subtotal (95% CI)**			**14**			**6**	**0.9%**	**4.60 [−3.81, 13.01]**
Heterogeneity: Not applicable								
Test for overall effect: Z = 1.07 (P = 0.28)								
**Total (95% CI)**			**385**			**354**	**100.0%**	**−1.04 [−1.84, −0.23]**
Heterogeneity: Tau² = 1.14; Chi² = 41.95, df = 11 (P < 0.0001); I² = 74%								
Test for overall effect: Z = 2.53 (P = 0.01)								
Test for subgroup differences: Chi² = 2.58, df = 2 (P = 0.28), I² = 22.4%								

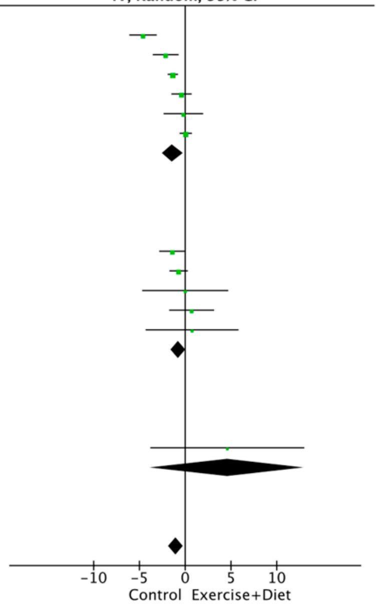

Figure S9: Subgroup analysis of the effects of diet/nutrition and physical activity/exercise intervention on insulin levels.

Study or Subgroup	Exercise+Diet Mean	SD	Total	Control Mean	SD	Total	Weight	Std. Mean Difference IV, Random, 95% CI
**1.22.1 Breast cancer**								
Karimi 2015 (breast)	5.1	1	10	7.4	1.3	10	7.3%	−1.90 [−2.99, −0.80]
D'Alonzo 2021 (breast)	13.2	9.6	55	18.1	10.6	51	25.7%	−0.48 [−0.87, −0.10]
Harrigan 2016 (breast)	15.67	10.88	64	18.31	9.19	33	24.1%	−0.25 [−0.67, 0.17]
**Subtotal (95% CI)**			**129**			**94**	**57.1%**	**−0.66 [−1.27, −0.05]**
Heterogeneity: Tau² = 0.20; Chi² = 7.56, df = 2 (P = 0.02); I² = 74%								
Test for overall effect: Z = 2.12 (P = 0.03)								
**1.22.2 Prostate cancer**								
Freedland 2019 (prostate)	262.06	157.59	11	333.18	269.39	18	12.9%	−0.30 [−1.05, 0.46]
Bourke 2011 (prostate)	8.91	8.4	21	11.7	14.2	21	16.9%	−0.23 [−0.84, 0.37]
Wilson 2021 (prostate)	12.66	12.59	14	13.33	11.11	14	13.2%	−0.05 [−0.80, 0.69]
**Subtotal (95% CI)**			**46**			**53**	**42.9%**	**−0.20 [−0.60, 0.20]**
Heterogeneity: Tau² = 0.00; Chi² = 0.22, df = 2 (P = 0.90); I² = 0%								
Test for overall effect: Z = 0.98 (P = 0.33)								
**Total (95% CI)**			**175**			**147**	**100.0%**	**−0.41 [−0.74, −0.08]**
Heterogeneity: Tau² = 0.07; Chi² = 9.01, df = 5 (P = 0.11); I² = 45%								
Test for overall effect: Z = 2.44 (P = 0.01)								
Test for subgroup differences: Chi² = 1.54, df = 1 (P = 0.22), I² = 34.9%								

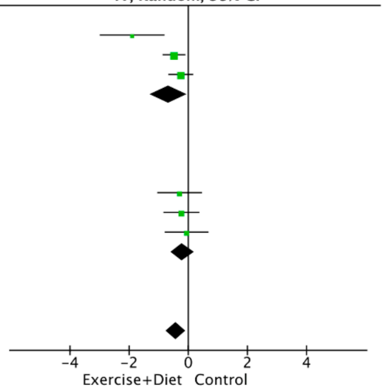

Figure S10: Subgroup analysis of the effects of diet/nutrition and physical activity/exercise intervention on glucose levels.

Study or Subgroup	Exercise+Diet Mean	SD	Total	Control Mean	SD	Total	Weight	Std. Mean Difference IV, Random, 95% CI
Reeves 2021 (breast)	5.33	0.6	67	5.6	0.59	57	29.7%	−0.45 [−0.81, −0.09]
Karimi 2015 (breast)	6.5	0.7	10	6.5	1	10	7.9%	0.00 [−0.88, 0.88]
Freedland 2019 (prostate)	98.47	17.44	11	97.44	20.27	18	10.3%	0.05 [−0.70, 0.80]
D'Alonzo 2021 (breast)	101	14	55	99.7	15.1	51	27.6%	0.09 [−0.29, 0.47]
Harrigan 2016 (breast)	104.61	23.44	64	102.39	13.56	33	24.5%	0.11 [−0.31, 0.53]
**Total (95% CI)**			**207**			**169**	**100.0%**	**−0.08 [−0.34, 0.18]**
Heterogeneity: Tau² = 0.03; Chi² = 5.77, df = 4 (P = 0.22); I² = 31%								
Test for overall effect: Z = 0.58 (P = 0.56)								

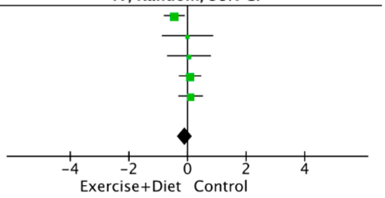

Figure S11: Subgroup analysis of the effects of diet/nutrition and physical activity/exercise intervention on HDL.

Study or Subgroup	Exercise+Diet Mean	SD	Total	Control Mean	SD	Total	Weight	Std. Mean Difference IV, Random, 95% CI
**1.28.1 Breast cancer**								
Scott 2013 (breast)	1.57	0.33	43	1.52	0.38	40	23.2%	0.14 [−0.29, 0.57]
Mefferd 2007 (breast)	62.3	13.5	47	57.9	14.5	29	21.1%	0.31 [−0.15, 0.78]
Reeves 2021 (breast)	1.45	0.19	67	1.39	0.19	57	28.8%	0.31 [−0.04, 0.67]
Karimi 2015 (breast)	53	5.6	10	44.5	5.1	10	6.1%	1.52 [0.50, 2.54]
**Subtotal (95% CI)**			**167**			**136**	**79.3%**	**0.38 [0.03, 0.73]**
Heterogeneity: Tau^2 = 0.06; Chi^2 = 5.97, df = 3 (P = 0.11); I^2 = 50%								
Test for overall effect: Z = 2.12 (P = 0.03)								
**1.28.2 Prostate cancer**								
Wilson 2021 (prostate)	1.3	0.4	14	1.3	0.5	14	10.7%	0.00 [−0.74, 0.74]
Freedland 2019 (prostate)	63.9	26.64	11	50.85	19.64	18	10.1%	0.56 [−0.20, 1.33]
**Subtotal (95% CI)**			**25**			**32**	**20.7%**	**0.27 [−0.28, 0.83]**
Heterogeneity: Tau^2 = 0.01; Chi^2 = 1.08, df = 1 (P = 0.30); I^2 = 7%								
Test for overall effect: Z = 0.97 (P = 0.33)								
**Total (95% CI)**			**192**			**168**	**100.0%**	**0.34 [0.07, 0.61]**
Heterogeneity: Tau^2 = 0.03; Chi^2 = 7.08, df = 5 (P = 0.22); I^2 = 29%								
Test for overall effect: Z = 2.49 (P = 0.01)								
Test for subgroup differences: Chi^2 = 0.10, df = 1 (P = 0.75), I^2 = 0%								

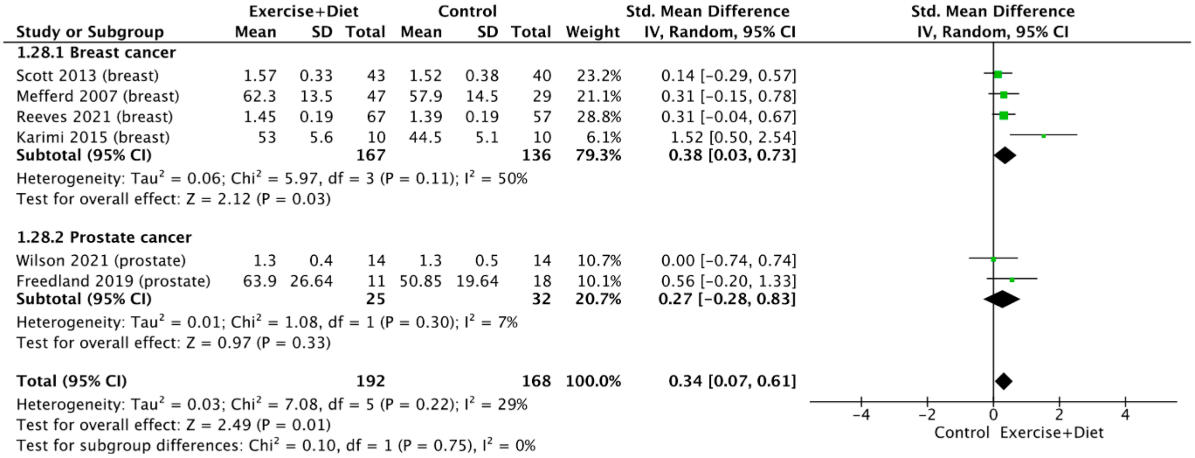

Figure S12: Forest plot of the effects of diet/nutrition and physical activity/exercise intervention on LDL.

Study or Subgroup	Exercise+Diet Mean	SD	Total	Control Mean	SD	Total	Weight	Std. Mean Difference IV, Random, 95% CI
Mefferd 2007 (breast)	102	32.2	47	116.5	45.3	29	48.5%	−0.38 [−0.85, 0.09]
Freedland 2019 (prostate)	118.3	47.67	11	136.97	51.33	18	18.5%	−0.36 [−1.12, 0.39]
Wilson 2021 (prostate)	2.6	1.2	14	2.8	1.4	14	19.2%	−0.15 [−0.89, 0.59]
Karimi 2015 (breast)	1.2	16	10	1.4	17.6	10	13.8%	−0.01 [−0.89, 0.87]
**Total (95% CI)**			**82**			**71**	**100.0%**	**−0.28 [−0.61, 0.04]**
Heterogeneity: Tau^2 = 0.00; Chi^2 = 0.71, df = 3 (P = 0.87); I^2 = 0%								
Test for overall effect: Z = 1.70 (P = 0.09)								

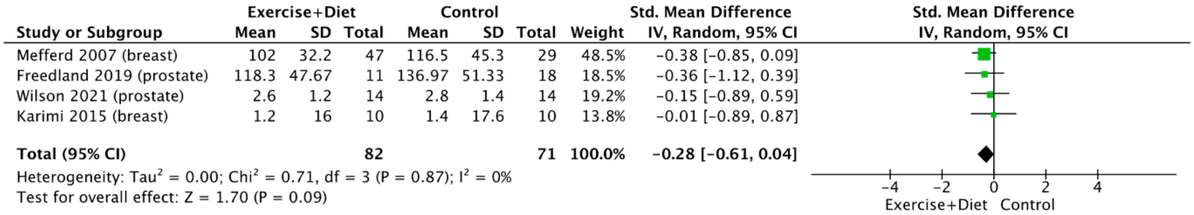

Figure S13: Subgroup analysis of the effects of diet/nutrition and physical activity/exercise intervention on triglycerides.

Study or Subgroup	Exercise+Diet Mean	SD	Total	Control Mean	SD	Total	Weight	Std. Mean Difference IV, Random, 95% CI
**1.31.1 Breast Cancer**								
Mefferd 2007 (breast)	134.8	74.2	47	175.4	80.6	29	26.3%	−0.52 [−0.99, −0.05]
Reeves 2021 (breast)	1.32	0.42	67	1.49	0.46	57	45.8%	−0.39 [−0.74, −0.03]
Karimi 2015 (breast)	1.16	13.9	10	1.38	22.6	10	7.6%	−0.01 [−0.89, 0.87]
**Subtotal (95% CI)**			**124**			**96**	**79.6%**	**−0.40 [−0.67, −0.13]**
Heterogeneity: Tau^2 = 0.00; Chi^2 = 1.03, df = 2 (P = 0.60); I^2 = 0%								
Test for overall effect: Z = 2.87 (P = 0.004)								
**1.31.2 Prostate Cancer**								
Freedland 2019 (prostate)	60.89	43.45	11	119.65	102.56	18	9.8%	−0.67 [−1.44, 0.11]
Wilson 2021 (prostate)	1.3	0.6	14	1.3	0.5	14	10.6%	0.00 [−0.74, 0.74]
**Subtotal (95% CI)**			**25**			**32**	**20.4%**	**−0.32 [−0.98, 0.33]**
Heterogeneity: Tau^2 = 0.07; Chi^2 = 1.49, df = 1 (P = 0.22); I^2 = 33%								
Test for overall effect: Z = 0.97 (P = 0.33)								
**Total (95% CI)**			**149**			**128**	**100.0%**	**−0.38 [−0.62, −0.14]**
Heterogeneity: Tau^2 = 0.00; Chi^2 = 2.58, df = 4 (P = 0.63); I^2 = 0%								
Test for overall effect: Z = 3.09 (P = 0.002)								
Test for subgroup differences: Chi^2 = 0.04, df = 1 (P = 0.84), I^2 = 0%								

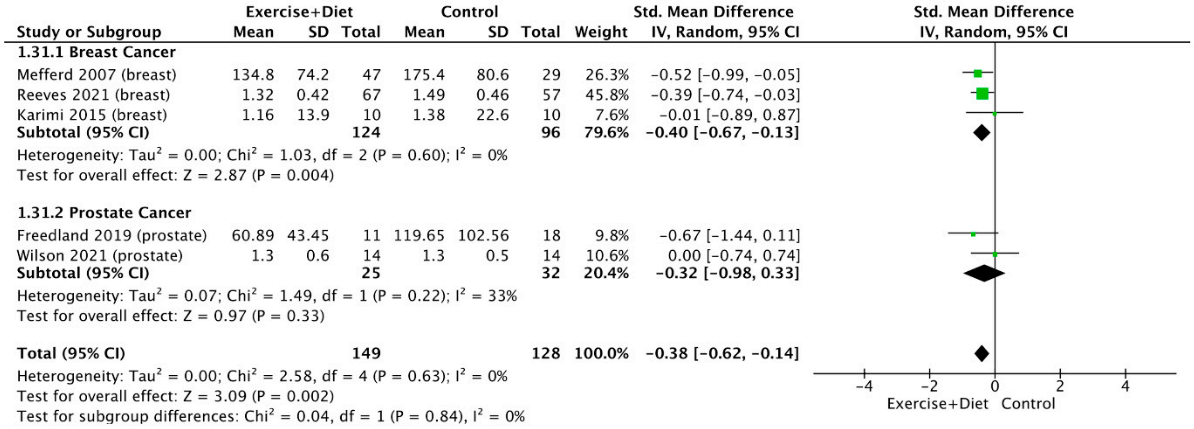

Figure S14: Forest plot of the effects of diet/nutrition and physical activity/exercise intervention on bone mineral density.

	Exercise+Diet			Control				Std. Mean Difference
Study or Subgroup	Mean	SD	Total	Mean	SD	Total	Weight	IV, Random, 95% CI
Harrigan 2016 (breast-in telephone)	1.1	0.03	24	1.159	0.04	31	24.8%	-1.62 [-2.23, -1.00]
Harrigan 2016 (breast)	1.129	0.03	30	1.159	0.04	31	25.2%	-0.84 [-1.36, -0.31]
Wilson 2021 (prostate)	2,576	291	14	2,567	278	14	24.2%	0.03 [-0.71, 0.77]
Brown 2021 (breast)	1.15	0.04	87	1.11	0.04	90	25.9%	1.00 [0.68, 1.31]
**Total (95% CI)**			**155**			**166**	**100.0%**	**-0.35 [-1.62, 0.93]**

Heterogeneity: Tau^2 = 1.61; Chi^2 = 73.05, df = 3 (P < 0.00001); I^2 = 96%
Test for overall effect: Z = 0.53 (P = 0.60)

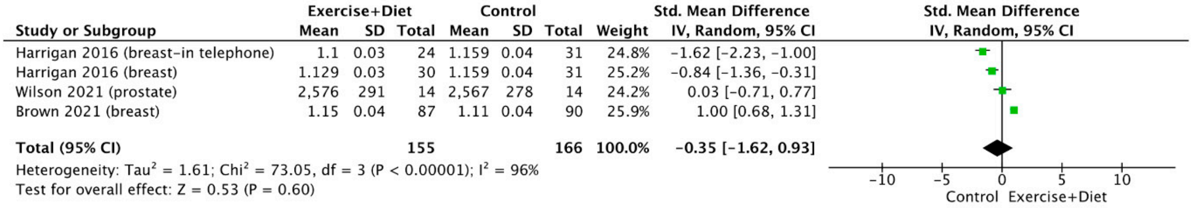

Figure S15: Forest plot of the effects of diet/nutrition and physical activity/exercise intervention on QoL mental health summary score.

	Exercise+Diet			Control				Std. Mean Difference
Study or Subgroup	Mean	SD	Total	Mean	SD	Total	Weight	IV, Random, 95% CI
Daubenmier 2006 (prostate)	50.7	9.3	40	56	6.7	42	11.8%	-0.65 [-1.10, -0.21]
Reeves 2016 (breast)	51.5	8.5	43	50.9	10.4	44	12.5%	0.06 [-0.36, 0.48]
Brown 2021a (breast)	74.29	15.67	87	71.94	16.22	90	16.5%	0.15 [-0.15, 0.44]
Morey 2009 (multiple)	86.1	9.47	319	84.26	13.28	322	21.3%	0.16 [0.00, 0.31]
Ferreira 2021 (lung)	68.17	22.1	18	63.9	19.1	8	5.2%	0.19 [-0.64, 1.03]
Ho 2020 (colorectal)	59.5	4.5	55	58	7.3	56	13.9%	0.25 [-0.13, 0.62]
Reeves 2021 (breast)	48.08	6.9	65	45.71	6.6	58	14.4%	0.35 [-0.01, 0.71]
Baguley 2022 (prostate)	55.2	4.36	11	50.3	4.4	10	4.4%	1.07 [0.15, 2.00]
**Total (95% CI)**			**638**			**630**	**100.0%**	**0.13 [-0.08, 0.35]**

Heterogeneity: Tau^2 = 0.05; Chi^2 = 17.83, df = 7 (P = 0.01); I^2 = 61%
Test for overall effect: Z = 1.19 (P = 0.23)

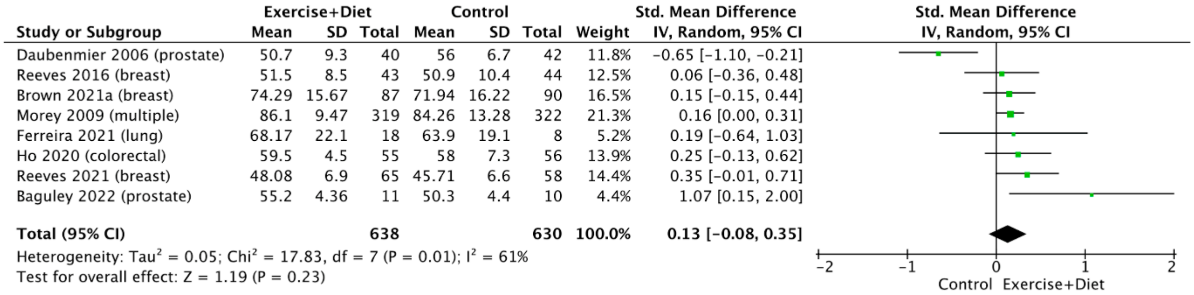

Figure S16: Forest plot of the effects of diet/nutrition and physical activity/exercise intervention on QoL social functioning.

	Exercise+Diet			Control				Std. Mean Difference
Study or Subgroup	Mean	SD	Total	Mean	SD	Total	Weight	IV, Random, 95% CI
Jacot 2020 (breast)	69.84	28.42	180	73.58	27.71	180	31.6%	-0.13 [-0.34, 0.07]
Ferreira 2021 (lung)	77.8	28.6	18	76.6	18.2	8	2.9%	0.04 [-0.79, 0.88]
Brown 2021a (breast)	82.02	18.84	87	81.04	18.97	90	18.8%	0.05 [-0.24, 0.35]
Morey 2009 (multiple)	88.91	18.75	319	85.75	21.89	322	44.4%	0.15 [-0.00, 0.31]
Baguley 2022 (prostate)	91.8	12.01	9	87.8	12.34	10	2.4%	0.31 [-0.59, 1.22]
**Total (95% CI)**			**613**			**610**	**100.0%**	**0.05 [-0.10, 0.19]**

Heterogeneity: Tau^2 = 0.01; Chi^2 = 5.08, df = 4 (P = 0.28); I^2 = 21%
Test for overall effect: Z = 0.62 (P = 0.54)

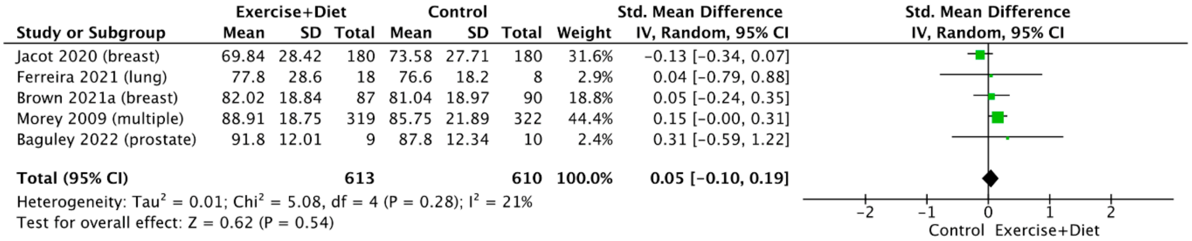

Figure S17: Forest plot of the effects of diet/nutrition and physical activity/exercise intervention on QoL bodily pain.

	Exercise+Diet			Control				Mean Difference
Study or Subgroup	Mean	SD	Total	Mean	SD	Total	Weight	IV, Random, 95% CI
Brown 2021a (breast)	72.43	17.53	87	72.88	18.5	90	25.2%	-0.45 [-5.76, 4.86]
Baguley 2022 (prostate)	77.8	19.29	11	76.8	19.04	10	2.6%	1.00 [-15.41, 17.41]
Morey 2009 (multiple)	71.42	19.11	319	69.41	21.89	322	70.2%	2.01 [-1.17, 5.19]
Ferreira 2021 (lung)	77.9	23.8	18	71.1	22.4	8	2.0%	6.80 [-12.22, 25.82]
**Total (95% CI)**			**435**			**430**	**100.0%**	**1.46 [-1.21, 4.12]**

Heterogeneity: Tau^2 = 0.00; Chi^2 = 0.92, df = 3 (P = 0.82); I^2 = 0%
Test for overall effect: Z = 1.07 (P = 0.28)

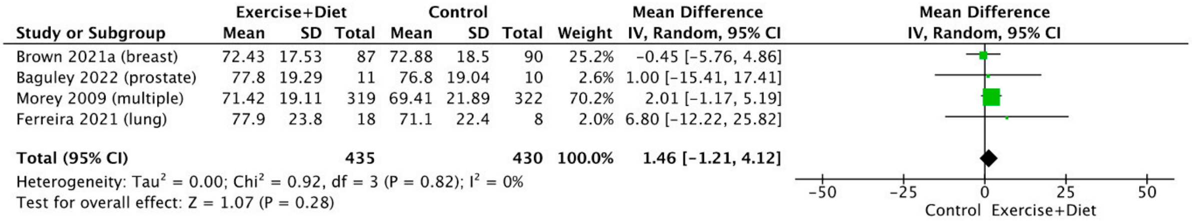

Figure S18: Subgroup analysis of the effects of diet/nutrition and physical activity/exercise intervention on QoL physical component summary score.

Study or Subgroup	Exercise+Diet Mean	SD	Total	Control Mean	SD	Total	Weight	Std. Mean Difference IV, Random, 95% CI
**1.11.1 Breast cancer**								
Reeves 2016 (breast)	49.5	88.8	43	49.1	10.5	44	6.5%	0.01 [−0.41, 0.43]
Reeves 2021 (breast)	47.96	6.9	65	46.4	6.6	58	9.1%	0.23 [−0.13, 0.58]
Brown 2021a (breast)	69.49	15.86	87	65.6	16.41	90	13.1%	0.24 [−0.06, 0.54]
**Subtotal (95% CI)**			**195**			**192**	**28.6%**	**0.18 [−0.02, 0.38]**
Heterogeneity: $Tau^2 = 0.00$; $Chi^2 = 0.89$, df = 2 (P = 0.64); $I^2 = 0\%$								
Test for overall effect: Z = 1.80 (P = 0.07)								
**1.11.2 Prostate cancer**								
Baguley 2022 (prostate)	48.7	6.09	11	49.6	6.13	10	1.6%	−0.14 [−1.00, 0.72]
O'Neil 2015 (prostate)	22.7	5.7	45	21.4	6.1	45	6.6%	0.22 [−0.20, 0.63]
Daubenmier 2006 (prostate)	53.2	6.6	40	50.2	9.5	42	6.0%	0.36 [−0.07, 0.80]
**Subtotal (95% CI)**			**96**			**97**	**14.2%**	**0.24 [−0.04, 0.52]**
Heterogeneity: $Tau^2 = 0.00$; $Chi^2 = 1.07$, df = 2 (P = 0.59); $I^2 = 0\%$								
Test for overall effect: Z = 1.65 (P = 0.10)								
**1.11.3 Other cancers**								
Morey 2009 (multiple)	73.75	16.07	319	70.76	16.15	322	47.4%	0.19 [0.03, 0.34]
Ferreira 2021 (lung)	67	20.1	18	61.8	21.2	8	1.6%	0.25 [−0.59, 1.08]
Ho 2020 (colorectal)	50.1	5.1	55	48.3	7.3	56	8.2%	0.28 [−0.09, 0.66]
**Subtotal (95% CI)**			**392**			**386**	**57.2%**	**0.20 [0.06, 0.34]**
Heterogeneity: $Tau^2 = 0.00$; $Chi^2 = 0.24$, df = 2 (P = 0.89); $I^2 = 0\%$								
Test for overall effect: Z = 2.79 (P = 0.005)								
**Total (95% CI)**			**683**			**675**	**100.0%**	**0.20 [0.09, 0.31]**
Heterogeneity: $Tau^2 = 0.00$; $Chi^2 = 2.29$, df = 8 (P = 0.97); $I^2 = 0\%$								
Test for overall effect: Z = 3.70 (P = 0.0002)								
Test for subgroup differences: $Chi^2 = 0.10$, df = 2 (P = 0.95), $I^2 = 0\%$								

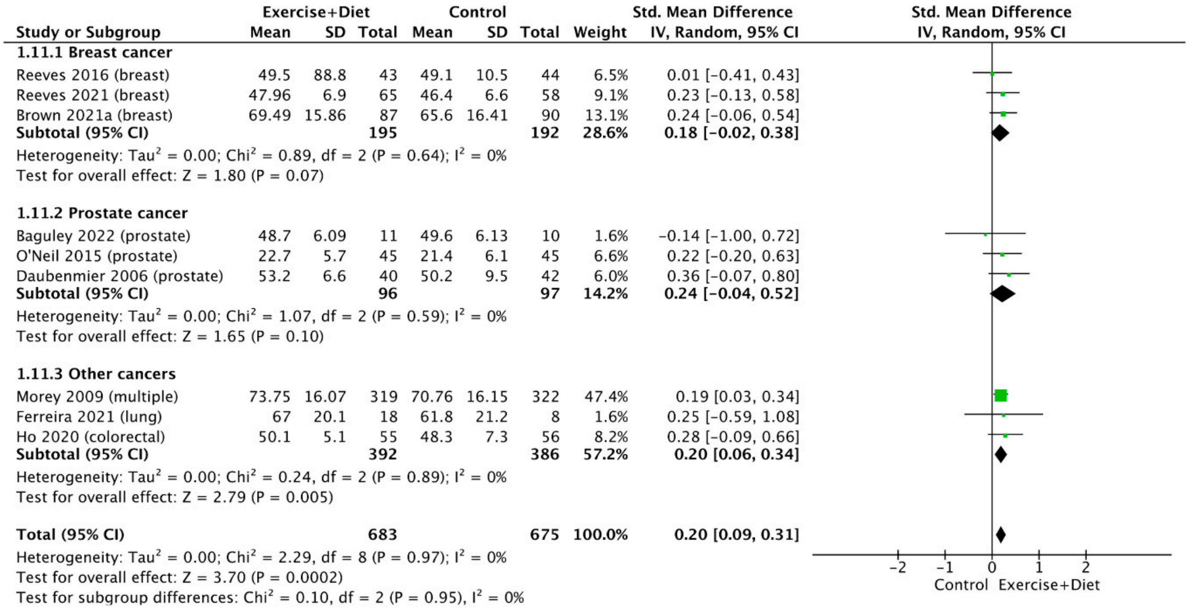

Figure S19: Subgroup analysis of the effects of diet/nutrition and physical activity/exercise intervention on QoL physical functioning.

Study or Subgroup	Exercise+Diet Mean	SD	Total	Control Mean	SD	Total	Weight	Std. Mean Difference IV, Random, 95% CI
**1.15.1 Breast cancer**								
Jacot 2020 (breast)	86.49	17.11	180	84.4	15.38	180	31.8%	0.13 [−0.08, 0.34]
Brown 2021a (breast)	74.03	16.04	87	69.36	16.41	90	15.5%	0.29 [−0.01, 0.58]
**Subtotal (95% CI)**			**267**			**270**	**47.2%**	**0.18 [0.01, 0.35]**
Heterogeneity: $Tau^2 = 0.00$; $Chi^2 = 0.74$, df = 1 (P = 0.39); $I^2 = 0\%$								
Test for overall effect: Z = 2.08 (P = 0.04)								
**1.15.2 Other cancers**								
Ferreira 2021 (lung)	59.4	26.5	18	69.4	26	8	1.9%	−0.37 [−1.21, 0.47]
Baguley 2022 (prostate)	78.3	15.99	11	82.2	16.05	10	1.8%	−0.23 [−1.09, 0.63]
Demark-Wahnefried 2012 (multiple)	74.4	17.88	269	70.5	18.7	289	49.0%	0.21 [0.05, 0.38]
**Subtotal (95% CI)**			**298**			**307**	**52.8%**	**0.08 [−0.26, 0.41]**
Heterogeneity: $Tau^2 = 0.03$; $Chi^2 = 2.67$, df = 2 (P = 0.26); $I^2 = 25\%$								
Test for overall effect: Z = 0.44 (P = 0.66)								
**Total (95% CI)**			**565**			**577**	**100.0%**	**0.18 [0.06, 0.29]**
Heterogeneity: $Tau^2 = 0.00$; $Chi^2 = 3.41$, df = 4 (P = 0.49); $I^2 = 0\%$								
Test for overall effect: Z = 2.99 (P = 0.003)								
Test for subgroup differences: $Chi^2 = 0.30$, df = 1 (P = 0.59), $I^2 = 0\%$								

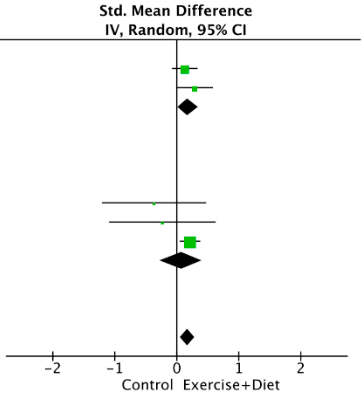

Supplement: Supplementary file 1 [file nutrients-16-01749-s001.zip › Supplement S1.pdf]
